# Top 100 Most-Cited Papers in Neuropathic Pain From 2000 to 2020: A Bibliometric Study

**DOI:** 10.3389/fneur.2021.765193

**Published:** 2021-11-12

**Authors:** Huan-Yu Xiong, Hao Liu, Xue-Qiang Wang

**Affiliations:** ^1^Department of Sport Rehabilitation, Shanghai University of Sport, Shanghai, China; ^2^Department of Rehabilitation, JORU Rehabilitation Hospital, Yixing, China

**Keywords:** neuropathic pain, citation analysis, bibliometric study, top-cited, web of science

## Abstract

**Background:** Neuropathic pain research has grown impressively in the past two decades, as evidenced by improvements in research quality and increments in the number of research papers. In views of this situation, the use of quantitative measurements to analyze and characterize existing research has become imperative. The aim of this research is to identify and analyze the 100 most-cited papers in neuropathic pain research.

**Methods:** Neuropathic pain-related articles published between 2000 and 2020 were screened from Web of Science (WOS) by using the following subject terms: TI = (Neuralgia$ OR Neurodynia$ OR “Neuropathic pain” OR sciatica OR “Nerve pain$”). The publications were ranked in a descending order on the basis of citation counts, and the top 100 most-cited neuropathic pain papers were determined. Subsequently, we conducted a bibliometric study to determine the authors, journals, countries, and institutions that contributed the most to the top 100 neuropathic pain lists; describe the keywords and hotspots of the top 100 most-cited papers; and explore the factors associated with successful citations.

**Results:** The top 100 most-cited papers were published from 2000 to 2017, and 2003 had the largest number of published papers (*n* = 16). The mean number of citations per paper was 480.72, with a range of 262–1,569. Forty-four kinds of journals contributed to the top 100 most-cited papers, which were predominantly published in “Pain” (*n* = 23). The USA was determined to be the leader of neuropathic pain research in terms of quality and quantity.

**Conclusion:** This study provides a comprehensive list of the most influential papers on neuropathic pain and demonstrates the important advances in this field to help understand academic concerns and the directions of technological innovations in neuropathic pain worldwide.

## Introduction

With the increase in neuropathic pain prevalence in the past years, neuropathic pain-related research has become a serious concern. Neuropathic pain, which is described as “pain caused by a lesion or disease of the somatosensory system (1),” is a major public health problem and has become a global burden (2, 3). Neuropathic pain can be caused by any nerve injury, with common etiologies such as diabetic polyneuropathy (4), trigeminal neuralgia (5), and sciatic nerve injury (6). Neuropathic pain can severely damage patients' quality of life, reduce work productivity, and cause disability in severe cases because it is usually related to other issues, such as dysfunction, anxiety, depression, and insomnia. Epidemiological studies have suggested that the prevalence of neuropathic pain could be between 6.9 and 10% (7, 8). The incidence of neuropathic pain exceeds 60% in patients with severe clinical neuropathy, and the cost increases annually. Moreover, the intensity and duration of neuropathic pain are higher than those of chronic pain without neuropathic characteristics (3, 9, 10). These findings indicate that the incidence of neuropathic pain is far from being low, and neuropathic pain is worth investigating. However, thus far, only a few studies have revealed the exact mechanism and treatment methods despite the increase in research on neuropathic pain (11).

Over the past 20 years, many experts and researchers have struggled to provide new insights into neuropathic pain, and numerous related study results have been published in various journals in the form of articles to clarify the underlying mechanisms and risk factors and explore the therapeutic targets of neuropathic pain (12–14). With the rapid accumulation of scientific literature on neuropathic pain, the most frequently cited papers are particularly important because high citations indicate high influence or visibility in the research community.

Bibliometrics is a research analysis based on the number and patterns of citations (15). Citation analysis in bibliometrics uses citation data, such as citation counts in a paper, to quantify the importance of a study. This approach can be utilized to explore various factors, including assessing the influence of publication (e.g., specific scientific community) or collections of research findings (e.g., all articles in a specific journal or study topic) (16). Several pain-related areas, such as headache (17), orofacial pain (18), and pediatric pain (19), have been measured and ranked in terms of research results in this manner, whether at the institution, country, or international level. Several recent publications have focused on bibliometric features of neuropathic pain research. For example, Yumeng Chen performed a bibliometric analysis of exercise and neuropathic pain research over a short period (2005–2019) (20), Jishi Ye mapped the publication trend of neuropathic pain in the world and China from 1998 to 2017 (21).

Through a literature search, we found that many papers on neuropathic pain have high citations (citation times > 200), but no analysis of the most influential works in the field of neuropathic pain has been conducted yet. Thus, we performed a bibliometric study to analyze the 100 most-cited papers in this field from 2000 to 2020 on the basis of the Web of Science (Thomson Reuters, USA, 2008) and determined the factors related to their successful citations. This task could be beneficial to the paper publication of investigators and design of future research. We look forward to identifying the most promising research directions for neuropathic pain.

## Methods

Ethical approval from an institutional review board was not required because our study was a bibliometric analysis that did not involve human subjects.

### Search Strategy

Neuropathic pain-related articles published between 2000 and 2020 were screened from the Science Citation Index–Expanded (SCI-Expanded) of Web of Science (WOS), which allows access to over 9,381 peer-reviewed journals that have been published in 178 scientific disciplines since 1945. All data were acquired on January 1, 2021, to avoid changes in the online activity of papers, and 9,561 results were produced. In the Science Citation Index–Expanded of WOS, the search terms were created in reference to several academic articles and MESH terms from PubMed, as follows: TI = (Neuralgia$ OR Neurodynia$ OR “Neuropathic pain” OR sciatica OR “Nerve pain$”) AND Language = English, with the period of 2000 to 2020.

### Inclusion Criteria

**Figure 1:**
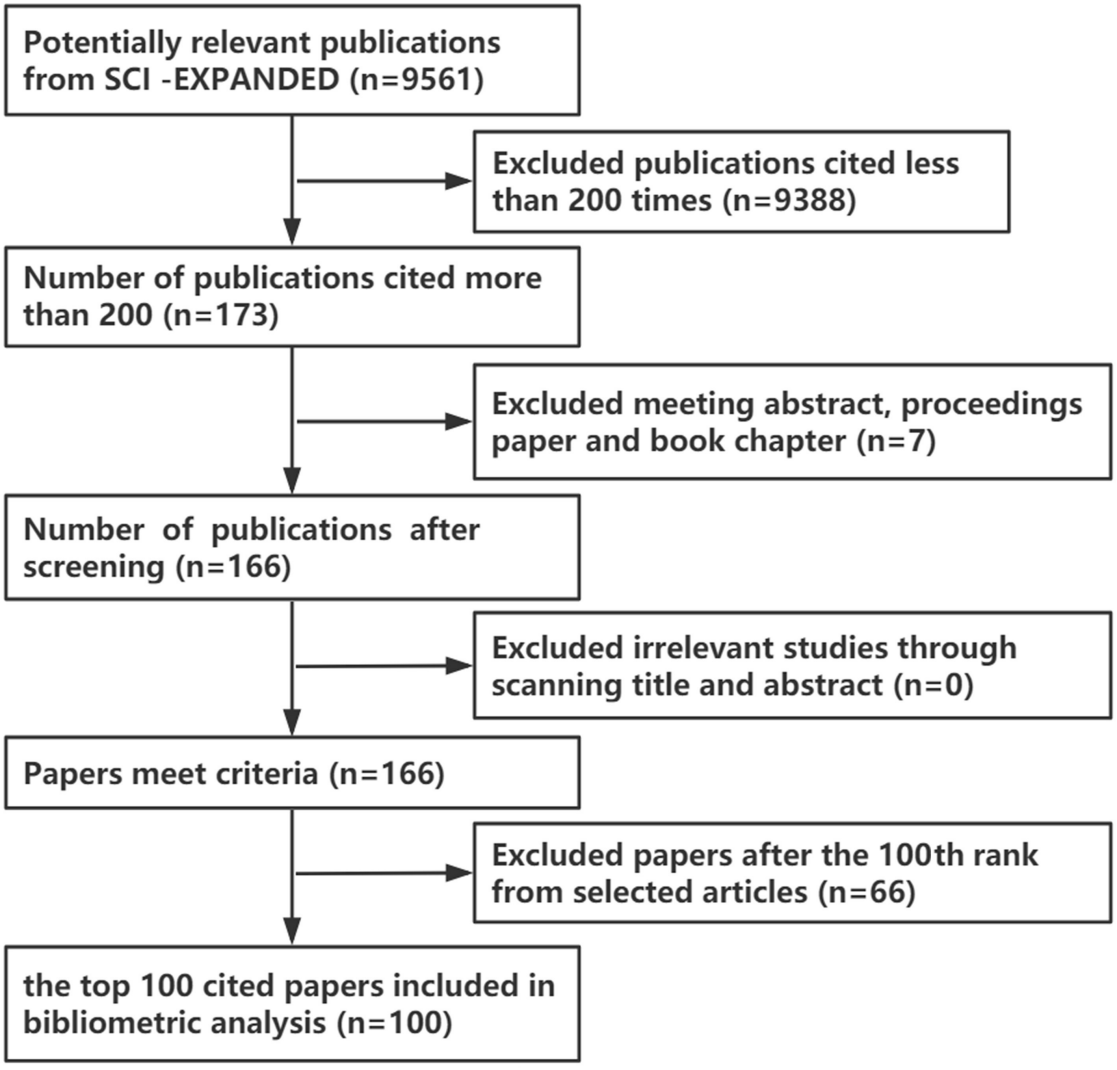
Data extraction process.

The publications were ranked in a descending order on the basis of citation counts and reviewed to determine the top 100 most-cited neuropathic pain papers. Papers with fewer than 200 citation times were excluded to reduce the number of papers that require follow-up screening. Figure 1 shows the selection process.

In terms of literature type, only articles and reviews were included; proceedings papers, conference abstracts, conference presentations, and book chapters were excluded. Two independent investigators reviewed the titles and abstracts and deleted studies that are not related to neuropathic pain. A total of 166 articles remained after reviewing the titles and abstracts. The 100 most-cited papers were then exported to Microsoft Excel 2016 to create tables and figures.

### Data Selection

Data selection and extraction were performed by two well-trained independent researchers (Huanyu Xiong and XueQiang Wang), and XueQiang Wang was consulted to deal with discrepancies. Related data, including publication date, citation counts, citation per year (total citations/the number of years since publication), author, journal of publication, country of origin (based on the correspondent author's address), institution, document type, research field, and keywords, were extracted from the top-cited papers and used to assess publication quality. In the search results from WOS, we screened several collections, such as journal, date, and study field. Then, we created citation reports and obtained data. The journal impact factor 2020 (IF 2020) and 5-year IF were from JCR 2020. A limitation of this study is that recent papers with high citation potential may not have been included because of the limited timespan since publication. To correct this limitation, we ran the same search within a short period (2018–2020) to select the top 10 most-cited papers.

### Statistical Methods

Statistical analyses were performed on the retrieved data by using SPSS Statistics 25.0 software. Statistical significance was set at *P* < 0.05. Descriptive statistics were quantified as the average or counts (percentages) of parameters. The Shapiro–Wilk test was applied to examine the normal distribution of individual variables. Data with a normal distribution were expressed as the mean ± standard deviation. A comparison of means was performed using the Mann–Whitney test was applied when necessary. The Pearson product moment correlation coefficient was employed to test the correlations between IF (2020) and paper counts, IF (2020) and citation counts, as well as correlations between annual citations and total citations. One-way analysis of variance (ANOVA) was performed to test qualitative indicators, including the distribution difference in paper count among country, type of document and open access before and after 2008. CiteSpace V was utilized to analyze and generate a network map of keyword co-occurrence (22).

## Results

### Distribution of Citations

We retrieved the 100 most frequently cited papers on neuropathic pain (Table 1), which received a total of 48,072 citations (WOS). The median value of the citations was 368 with a range of 262–1,569. For annual citations, the median value was 25.47 with a range of 13.6–206.67. Nine papers were cited more than 1,000 times, and 21 were cited more than 500 times. The article entitled “Neuropathic pain–Redefinition and grading system for clinical and research purposes” from Treede, R.D., was the most-cited publication (*n* = 1,569) (23). Since the article's publication in 2008, the number of citations has been increasing yearly, reaching 120.69 per year. The papers with high annual citations had high total citations, and the correlation between these results was significantly strong (*r* = 0.795, *P* = 0.000).

**Table 1:** The 100 most-cited papers in Neuropathic pain field.

**Rank**	**First author**	**Paper**	**Citations WOS**	**Citations per year**	**Citations in 2020**
1	Treede, RD	Treede R-D, Jensen T S, Campbell J N, et al. Neuropathic pain: redefinition and a grading system for clinical and research purposes [J]. Neurology, 2008, 70: 1630-5.	1,569	120.69	106
2	Oxman, MN	Oxman M N, Levin M J, Johnson G R, et al. A vaccine to prevent herpes zoster and postherpetic neuralgia in older adults [J]. N Engl J Med, 2005, 352: 2271-84.	1,449	90.56	59
3	Rolke, R	Rolke R, Baron R, Maier C, et al. Quantitative sensory testing in the German Research Network on Neuropathic Pain (DFNS): standardized protocol and reference values [J]. Pain, 2006, 123: 231-243.	1,259	83.93	126
4	Finnerup, NB	Finnerup Nanna B, Attal Nadine, Haroutounian Simon, et al. Pharmacotherapy for neuropathic pain in adults: a systematic review and meta-analysis [J]. Lancet Neurol, 2015, 14: 162-73.	1,240	206.67	235
5	Dworkin, RH	Dworkin Robert H, O'Connor Alec B, Backonja Miroslav, et al. Pharmacologic management of neuropathic pain: evidence-based recommendations [J]. Pain, 2007, 132: 237-251.	1,232	88	56
6	Decosterd, I	Decosterd Isabelle, Woolf Clifford J, Spared nerve injury: an animal model of persistent peripheral neuropathic pain [J]. Pain, 2000, 87: 149-158.	1,219	58.05	99
7	Coull, JAM	Coull Jeffrey A M, Beggs Simon, Boudreau Dominic, et al. BDNF from microglia causes the shift in neuronal anion gradient underlying neuropathic pain [J]. Nature, 2005, 438: 1017-21.	1,187	74.19	93
8	Bouhassira, D	Bouhassira Didier, Attal Nadine, Alchaar Haiel, et al. Comparison of pain syndromes associated with nervous or somatic lesions and development of a new neuropathic pain diagnostic questionnaire (DN4) [J]. Pain, 2005, 114: 29-36.	1,140	71.25	111
9	Scholz, J	Scholz Joachim, Woolf Clifford J, The neuropathic pain triad: neurons, immune cells and glia [J]. Nat Neurosci, 2007, 10: 1361-8.	1,087	77.64	74
10	Attal, N	Attal N, Cruccu G, Baron R, et al. EFNS guidelines on the pharmacological treatment of neuropathic pain: 2010 revision [J]. Eur J Neurol, 2010, 17: 1113-e88.	997	90.64	82
11	Finnerup, NB	Finnerup N B, Otto M, McQuay H J, et al. Algorithm for neuropathic pain treatment: an evidence based proposal [J]. Pain, 2005, 118: 289-305.	783	48.94	13
12	Dworkin, RH	Dworkin Robert H, O'Connor Alec B, Audette Joseph, et al. Recommendations for the pharmacological management of neuropathic pain: an overview and literature update [J]. Mayo Clin Proc, 2010, 85: S3-14.	772	70.18	52
13	Dworkin, RH	Dworkin Robert H, Backonja Miroslav, Rowbotham Michael C, et al. Advances in neuropathic pain: diagnosis, mechanisms, and treatment recommendations [J]. Arch Neurol, 2003, 60: 1524-34.	753	41.83	8
14	Baron, R	Baron Ralf, Binder Andreas, Wasner Gunnar, Neuropathic pain: diagnosis, pathophysiological mechanisms, and treatment [J]. Lancet Neurol, 2010, 9: 807-19.	746	67.82	73
15	Campbell, JN	Campbell James N, Meyer Richard A, Mechanisms of neuropathic pain [J]. Neuron, 2006, 52: 77-92.	718	47.87	61
16	Coull, JAM	Coull Jeffrey A M, Boudreau Dominic, Bachand Karine, et al. Trans-synaptic shift in anion gradient in spinal lamina I neurons as a mechanism of neuropathic pain [J]. Nature, 2003, 424: 938-42.	697	38.72	38
17	Gilron, I	Gilron Ian, Bailey Joan M, Tu Dongsheng, et al. Morphine, gabapentin, or their combination for neuropathic pain [J]. N Engl J Med, 2005, 352: 1324-34.	664	41.5	16
18	Jin, SX	Jin Shan-Xue, Zhuang Zhi-Ye, Woolf Clifford J, et al. p38 mitogen-activated protein kinase is activated after a spinal nerve ligation in spinal cord microglia and dorsal root ganglion neurons and contributes to the generation of neuropathic pain [J]. J Neurosci, 2003, 23: 4017-22.	635	35.28	30
19	Bouhassira, D	Bouhassira Didier, Attal Nadine, Fermanian Jacques, et al. Development and validation of the Neuropathic Pain Symptom Inventory [J]. Pain, 2004, 108: 248-257.	626	36.82	62
20	Finnerup, NB	Finnerup Nanna Brix, Sindrup Søren Hein, Jensen Troels Staehelin, The evidence for pharmacological treatment of neuropathic pain [J]. Pain, 2010, 150: 573-581.	614	55.82	35
21	Haanpaa, M	Haanpää Maija, Attal Nadine, Backonja Miroslav, et al. NeuPSIG guidelines on neuropathic pain assessment [J]. Pain, 2011, 152: 14-27.	587	58.7	42
22	Tsuda, M	Tsuda Makoto, Inoue Kazuhide, Salter Michael W, Neuropathic pain and spinal microglia: a big problem from molecules in “small” glia [J]. Trends Neurosci, 2005, 28: 101-7.	583	36.44	16
23	Chessell, IP	Chessell Iain P, Hatcher Jonathan P, Bountra Chas, et al. Disruption of the P2X7 purinoceptor gene abolishes chronic inflammatory and neuropathic pain [J]. Pain, 2005, 114: 386-396.	554	34.63	24
24	Kumar, K	Kumar Krishna, Taylor Rod S, Jacques Line, et al. Spinal cord stimulation vs. conventional medical management for neuropathic pain: a multicentre randomized controlled trial in patients with failed back surgery syndrome [J]. Pain, 2007, 132: 179-88.	552	39.43	61
25	Zhuang, ZY	Zhuang Zhi-Ye, Gerner Peter, Woolf Clifford J, et al. ERK is sequentially activated in neurons, microglia, and astrocytes by spinal nerve ligation and contributes to mechanical allodynia in this neuropathic pain model [J]. Pain, 2005, 114: 149-59.	550	50	42
26	Maier, C	Maier C, Baron R, Tölle T R, et al. Quantitative sensory testing in the German Research Network on Neuropathic Pain (DFNS): somatosensory abnormalities in 1236 patients with different neuropathic pain syndromes [J]. Pain, 2010, 150: 439-450.	550	34.38	26
27	Dworkin, RH	Dworkin R H, Corbin A E, Young J P, et al. Pregabalin for the treatment of postherpetic neuralgia: a randomized, placebo-controlled trial [J]. Neurology, 2003, 60: 1274-83.	534	28.67	13
28	Attal, N	Attal N, Cruccu G, Baron R, et al. EFNS guidelines on the pharmacological treatment of neuropathic pain: 2010 revision [J]. Eur J Neurol, 2010, 17: 1113-e88.	529	35.27	10
29	Milligan, ED	Milligan Erin D, Twining Carin, Chacur Marucia, et al. Spinal glia and proinflammatory cytokines mediate mirror-image neuropathic pain in rats [J]. J Neurosci, 2003, 23: 1026-40.	523	29.06	11
30	Moalem, G	Moalem Gila, Tracey David J, Immune and inflammatory mechanisms in neuropathic pain [J]. Brain Res Rev, 2006, 51: 240-64.	513	34.2	36
31	Forero, M	Forero Mauricio, Adhikary Sanjib D, Lopez Hector, et al. The Erector Spinae Plane Block: A Novel Analgesic Technique in Thoracic Neuropathic Pain [J]. Reg Anesth Pain Med, 2016, 41: 621-7.	489	97.8	171
32	Kawasaki, Y	Kawasaki Yasuhiko, Xu Zhen-Zhong, Wang Xiaoying, et al. Distinct roles of matrix metalloproteases in the early- and late-phase development of neuropathic pain [J]. Nat Med, 2008, 14: 331-6.	472	36.31	51
33	Baron, R	Baron Ralf, Mechanisms of disease: neuropathic pain–a clinical perspective [J]. Nat Clin Pract Neurol, 2006, 2: 95-106.	454	30.27	21
34	van Hecke, O	van Hecke O, Austin Sophie K, Khan Rafi A, et al. Neuropathic pain in the general population: a systematic review of epidemiological studies [J]. Pain, 2014, 155: 654-662.	451	64.43	116
35	O'Connor, AB	O'Connor Alec B, Dworkin Robert H, Treatment of neuropathic pain: an overview of recent guidelines [J]. Am J Med, 2009, 122: S22-32.	451	37.58	11
36	Abbadie, C	Abbadie Catherine, Lindia Jill A, Cumiskey Anne Marie, et al. Impaired neuropathic pain responses in mice lacking the chemokine receptor CCR2 [J]. Proc Natl Acad Sci U S A, 2003, 100: 7947-52.	438	24.33	16
37	von Hehn, CA	von Hehn Christian A, Baron Ralf, Woolf Clifford J, Deconstructing the neuropathic pain phenotype to reveal neural mechanisms [J]. Neuron, 2012, 73: 638-52.	437	48.56	33
38	Rice, ASC	Rice A S C, Maton S, Postherpetic Neuralgia Study Group, Gabapentin in postherpetic neuralgia: a randomized, double blind, placebo controlled study [J]. Pain, 2001, 94: 215-224.	424	21.2	6
39	Love, S	Love S, Coakham H B, Trigeminal neuralgia: pathology and pathogenesis [J]. Brain, 2001, 124: 2347-60.	407	20.35	31
40	Boucher, TJ	Boucher T J, Okuse K, Bennett D L, et al. Potent analgesic effects of GDNF in neuropathic pain states [J]. Science, 2000, 290: 124-7.	402	19.14	13
41	Colloca, L	Colloca Luana, Ludman Taylor, Bouhassira Didier, et al. Neuropathic pain [J]. Nat Rev Dis Primers, 2017, 3: 17002.	387	96.75	171
42	Xiao, HS	Xiao Hua-Sheng, Huang Qiu-Hua, Zhang Fang-Xiong, et al. Identification of gene expression profile of dorsal root ganglion in the rat peripheral axotomy model of neuropathic pain [J]. Proc Natl Acad Sci U S A, 2002, 99: 8360-5.	385	25.67	18
43	Zhuang, ZY	Zhuang Zhi-Ye, Wen Yeong-Ray, Zhang De-Ren, et al. A peptide c-Jun N-terminal kinase (JNK) inhibitor blocks mechanical allodynia after spinal nerve ligation: respective roles of JNK activation in primary sensory neurons and spinal astrocytes for neuropathic pain development and maintenance [J]. J Neurosci, 2006, 26: 3551-60.	385	20.26	16
44	Freynhagen, R	Freynhagen Rainer, Strojek Krzysztof, Griesing Teresa, et al. Efficacy of pregabalin in neuropathic pain evaluated in a 12-week, randomized, double-blind, multicentre, placebo-controlled trial of flexible- and fixed-dose regimens [J]. Pain, 2005, 115: 254-263.	383	23.94	10
45	Cruccu, G	Cruccu G, Aziz T Z, Garcia-Larrea L, et al. EFNS guidelines on neurostimulation therapy for neuropathic pain [J]. Eur J Neurol, 2007, 14: 952-70.	380	27.14	14
46	Peul, WC	Peul Wilco C, van Houwelingen Hans C, van den Hout Wilbert B, et al. Surgery vs. prolonged conservative treatment for sciatica [J]. N Engl J Med, 2007, 356: 2245-56.	378	27	33
47	Ji, RR	Ji Ru-Rong, Suter Marc R, p38 MAPK, microglial signaling, and neuropathic pain [J]. Mol Pain, 2007, 3: 33.	373	26.64	28
48	Inoue, M	Inoue Makoto, Rashid Md Harunor, Fujita Ryousuke, et al. Initiation of neuropathic pain requires lysophosphatidic acid receptor signaling [J]. Nat Med, 2004, 10: 712-8.	372	21.88	23
49	Schmader, KE	Schmader Kenneth E, Epidemiology and impact on quality of life of postherpetic neuralgia and painful diabetic neuropathy [J]. Clin J Pain, 2002, 18: 350-4.	371	19.53	10
50	Gao, YJ	Gao Yong-Jing, Ji Ru-Rong, Chemokines, neuronal-glial interactions, and central processing of neuropathic pain [J]. Pharmacol Ther, 2010, 126: 56-68.	365	33.18	29
51	Raja, SN	Raja S N, Haythornthwaite J A, Pappagallo M, et al. Opioids vs. antidepressants in postherpetic neuralgia: a randomized, placebo-controlled trial [J]. Neurology, 2002, 59: 1015-21.	365	19.21	9
52	Ibrahim, MM	Ibrahim Mohab M, Deng Hongfeng, Zvonok Alexander, et al. Activation of CB2 cannabinoid receptors by AM1241 inhibits experimental neuropathic pain: pain inhibition by receptors not present in the CNS [J]. Proc Natl Acad Sci U S A, 2003, 100: 10529-33.	363	25.93	4
53	Moulin, D	Moulin Dwight, Boulanger Aline, Clark A J, et al. Pharmacological management of chronic neuropathic pain: revised consensus statement from the Canadian Pain Society [J]. Pain Res Manag, 2014, 19: 328-35.	363	20.17	16
54	Sabatowski, R	Sabatowski Rainer, Gálvez Rafael, Cherry David A, et al. Pregabalin reduces pain and improves sleep and mood disturbances in patients with post-herpetic neuralgia: results of a randomized, placebo-controlled clinical trial [J]. Pain, 2004, 109: 26-35.	362	21.29	9
55	Gao, YJ	Gao Yong-Jing, Zhang Ling, Samad Omar Abdel, et al. JNK-induced MCP-1 production in spinal cord astrocytes contributes to central sensitization and neuropathic pain [J]. J Neurosci, 2009, 29: 4096-108.	361	30.08	31
56	Austin, PJ	Austin Paul J, Moalem-Taylor Gila, The neuro-immune balance in neuropathic pain: involvement of inflammatory immune cells, immune-like glial cells and cytokines [J]. J Neuroimmunol, 2010, 229: 26-50.	355	32.27	38
57	Watson, CPN	Watson C Peter N, Moulin Dwight, Watt-Watson Judith, et al. Controlled-release oxycodone relieves neuropathic pain: a randomized controlled trial in painful diabetic neuropathy [J]. Pain, 2003, 105: 71-8.	343	19.06	7
58	Jarvis, MF	Jarvis Michael F, Burgard Edward C, McGaraughty Steve, et al. A-317491, a novel potent and selective non-nucleotide antagonist of P2X3 and P2X2/3 receptors, reduces chronic inflammatory and neuropathic pain in the rat [J]. Proc Natl Acad Sci U S A, 2002, 99: 17179-84.	341	21.31	10
59	Raskin, J	Raskin Joel, Pritchett Yili L, Wang Fujun, et al. A double-blind, randomized multicenter trial comparing duloxetine with placebo in the management of diabetic peripheral neuropathic pain [J]. Pain Med, 2005, 6: 346-56.	341	17.95	15
60	Kumar, K	Kumar Krishna, Taylor Rod S, Jacques Line, et al. The effects of spinal cord stimulation in neuropathic pain are sustained: a 24-month follow-up of the prospective randomized controlled multicenter trial of the effectiveness of spinal cord stimulation [J]. Neurosurgery, 2008, 63: 762-70; discussion 770.	339	26.08	32
61	Jensen, MP	Jensen Mark P, Chodroff Marci J, Dworkin Robert H, The impact of neuropathic pain on health-related quality of life: review and implications [J]. Neurology, 2007, 68: 1178-82.	336	24	25
62	Cruccu, G	Cruccu G, Anand P, Attal N, et al. EFNS guidelines on neuropathic pain assessment [J]. Eur J Neurol, 2004, 11: 153-62.	335	23.93	16
63	Bennett, MI	Bennett Michael I, Attal Nadine, Backonja Miroslav M, et al. Using screening tools to identify neuropathic pain [J]. Pain, 2007, 127: 199-203.	335	19.71	2
64	Jensen, TS	Jensen Troels S, Baron Ralf, Translation of symptoms and signs into mechanisms in neuropathic pain [J]. Pain, 2003, 102: 1-8.	334	18.56	11
65	Saarto, T	Saarto T, Wiffen P J, Antidepressants for neuropathic pain [J]. Cochrane Database Syst Rev, 2005, undefined: CD005454.	330	23.57	12
66	Saarto, T	Saarto T, Wiffen P J, Antidepressants for neuropathic pain [J]. Cochrane Database Syst Rev, 2007, undefined: CD005454.	330	20.63	12
67	Leung, L	Leung Lawrence, Cahill Catherine M, TNF-alpha and neuropathic pain–a review [J]. J Neuroinflammation, 2010, 7: 27.	329	29.91	36
68	Lai, J	Lai Josephine, Gold Michael S, Kim Chang Sook, et al. Inhibition of neuropathic pain by decreased expression of the tetrodotoxin-resistant sodium channel, NaV1.8 [J]. Pain, 2002, 95: 143-52.	327	17.21	6
69	Wernicke, JF	Wernicke J F, Pritchett Y L, D'Souza D N, et al. A randomized controlled trial of duloxetine in diabetic peripheral neuropathic pain [J]. Neurology, 2006, 67: 1411-20.	317	26.42	26
70	King, T	King Tamara, Vera-Portocarrero Louis, Gutierrez Tannia, et al. Unmasking the tonic-aversive state in neuropathic pain [J]. Nat Neurosci, 2009, 12: 1364-6.	317	21.13	10
71	Ulmann, L	Ulmann Lauriane, Hatcher Jon P, Hughes Jane P, et al. Up-regulation of P2X4 receptors in spinal microglia after peripheral nerve injury mediates BDNF release and neuropathic pain [J]. J Neurosci, 2008, 28: 11263-8.	316	24.31	32
72	Rowbotham, MC	Rowbotham Michael C, Twilling Lisa, Davies Pamela S, et al. Oral opioid therapy for chronic peripheral and central neuropathic pain [J]. N Engl J Med, 2003, 348: 1223-32.	313	17.39	6
73	Kim, HK	Kim Hee Kee, Park Soon Kwon, Zhou Jun-Li, et al. Reactive oxygen species (ROS) play an important role in a rat model of neuropathic pain [J]. Pain, 2004, 111: 116-24.	300	17.65	22
74	Clark, AK	Clark Anna K, Yip Ping K, Grist John, et al. Inhibition of spinal microglial cathepsin S for the reversal of neuropathic pain [J]. Proc Natl Acad Sci U S A, 2007, 104: 10655-60.	299	23	24
75	Cruccu, G	Cruccu G, Gronseth G, Alksne J, et al. AAN-EFNS guidelines on trigeminal neuralgia management [J]. Eur J Neurol, 2008, 15: 1013-28.	299	21.36	19
76	Finnerup, NB	Finnerup Nanna B, Haroutounian Simon, Kamerman Peter, et al. Neuropathic pain: an updated grading system for research and clinical practice [J]. Pain, 2016, 157: 1599-1606.	297	59.4	92
77	Dorn, G	Dorn Gabriele, Patel Sadhana, Wotherspoon Glen, et al. siRNA relieves chronic neuropathic pain [J]. Nucleic Acids Res, 2004, 32: e49.	294	17.29	1
78	Fox, A	Fox A, Kesingland A, Gentry C, et al. The role of central and peripheral Cannabinoid1 receptors in the antihyperalgesic activity of cannabinoids in a model of neuropathic pain [J]. Pain, 2001, 92: 91-100.	290	14.5	8
79	Zhang, J	Zhang Ji, Shi Xiang Qun, Echeverry Stefania, et al. Expression of CCR2 in both resident and bone marrow-derived microglia plays a critical role in neuropathic pain [J]. J Neurosci, 2007, 27: 12396-406.	289	20.64	16
80	Carragee, EJ	Carragee Eugene J, Han Michael Y, Suen Patrick W, et al. Clinical outcomes after lumbar discectomy for sciatica: the effects of fragment type and anular competence [J]. J Bone Joint Surg Am, 2003, 85: 102-8.	288	16	30
81	Walker, KM	Walker Katharine M, Urban Laszlo, Medhurst Stephen J, et al. The VR1 antagonist capsazepine reverses mechanical hyperalgesia in models of inflammatory and neuropathic pain [J]. J Pharmacol Exp Ther, 2003, 304: 56-62.	286	15.89	5
82	Gold, MS	Gold Michael S, Weinreich Daniel, Kim Chang-Sook, et al. Redistribution of Na(V)1.8 in uninjured axons enables neuropathic pain [J]. J Neurosci, 2003, 23: 158-66.	283	18.87	18
83	Honore, P	Honore Prisca, Donnelly-Roberts Diana, Namovic Marian T, et al. A-740003 [N-(1-{[(cyanoimino)(5-quinolinylamino) methyl]amino}G-2, 2-dimethylpropyl)-2-(3, 4-dimethoxyphenyl)acetamide], a novel and selective P2X7 receptor antagonist, dose-dependently reduces neuropathic pain in the rat [J]. J Pharmacol Exp Ther, 2006, 319: 1376-85.	283	17.69	15
84	Sindrup, SH	Sindrup Søren H, Otto Marit, Finnerup Nanna B, et al. Antidepressants in the treatment of neuropathic pain [J]. Basic Clin Pharmacol Toxicol, 2005, 96: 399-409.	283	15.72	5
85	Luo, ZD	Luo Z D, Calcutt N A, Higuera E S, et al. Injury type-specific calcium channel alpha 2 delta-1 subunit up-regulation in rat neuropathic pain models correlates with antiallodynic effects of gabapentin [J]. J Pharmacol Exp Ther, 2002, 303: 1199-205.	282	16.59	11
86	Verge, GM	Verge Gail M, Milligan Erin D, Maier Steve F, et al. Fractalkine (CX3CL1) and fractalkine receptor (CX3CR1) distribution in spinal cord and dorsal root ganglia under basal and neuropathic pain conditions [J]. Eur J Neurosci, 2004, 20: 1150-60.	282	14.84	11
87	Tanga, FY	Tanga F Y, Raghavendra V, DeLeo J A, Quantitative real-time RT-PCR assessment of spinal microglial and astrocytic activation markers in a rat model of neuropathic pain [J]. Neurochem Int, 2004, 45: 397-407.	281	16.53	12
88	Sweitzer, S	Sweitzer S, Martin D, DeLeo J A, Intrathecal interleukin-1 receptor antagonist in combination with soluble tumor necrosis factor receptor exhibits an anti-allodynic action in a rat model of neuropathic pain [J]. Neuroscience, 2001, 103: 529-39.	279	13.95	6
89	Sung, B	Sung Backil, Lim Grewo, Mao Jianren, Altered expression and uptake activity of spinal glutamate transporters after nerve injury contribute to the pathogenesis of neuropathic pain in rats [J]. J Neurosci, 2003, 23: 2899-910.	278	25.27	11
90	Cruccu, G	Cruccu G, Sommer C, Anand P, et al. EFNS guidelines on neuropathic pain assessment: revised 2009 [J]. Eur J Neurol, 2010, 17: 1010-8.	278	15.44	8
91	Bridges, D	Bridges D, Thompson S W, Rice A S, Mechanisms of neuropathic pain [J]. Br J Anaesth, 2001, 87: 12-26.	272	13.6	6
92	Khedr, EM	Khedr E M, Kotb H, Kamel N F, et al. Longlasting antalgic effects of daily sessions of repetitive transcranial magnetic stimulation in central and peripheral neuropathic pain [J]. J Neurol Neurosurg Psychiatry, 2005, 76: 833-8.	271	16.94	11
93	Proudfoot, CJ	Proudfoot Clare J, Garry Emer M, Cottrell David F, et al. Analgesia mediated by the TRPM8 cold receptor in chronic neuropathic pain [J]. Curr Biol, 2006, 16: 1591-605.	269	17.93	13
94	Gronseth, G	Gronseth G, Cruccu G, Alksne J, et al. Practice parameter: the diagnostic evaluation and treatment of trigeminal neuralgia (an evidence-based review): report of the Quality Standards Subcommittee of the American Academy of Neurology and the European Federation of Neurological Societies [J]. Neurology, 2008, 71: 1183-90.	265	20.38	24
95	Backonja, M	Backonja Miroslav, Glanzman Robert L, Gabapentin dosing for neuropathic pain: evidence from randomized, placebo-controlled clinical trials [J]. Clin Ther, 2003, 25: 81-104.	265	14.72	7
96	Eisenberg, E	Eisenberg Elon, McNicol Ewan D, Carr Daniel B, Efficacy and safety of opioid agonists in the treatment of neuropathic pain of nonmalignant origin: systematic review and meta-analysis of randomized controlled trials [J]. JAMA, 2005, 293: 3043-52.	264	16.5	10
97	Guindon, J	Guindon J, Hohmann A G, Cannabinoid CB2 receptors: a therapeutic target for the treatment of inflammatory and neuropathic pain [J]. Br J Pharmacol, 2008, 153: 319-34.	263	20.23	18
98	Krause, SJ	Krause Steven J, Backonja Misha-Miroslav, Development of a neuropathic pain questionnaire [J]. Clin J Pain, 2003, 19: 306-14.	263	14.61	12
99	Gilron, I	Gilron Ian, Bailey Joan M, Tu Dongsheng, et al. Nortriptyline and gabapentin, alone and in combination for neuropathic pain: a double-blind, randomized controlled crossover trial [J]. Lancet, 2009, 374: 1252-61.	262	21.83	11
100	Siddall, PJ	Siddall P J, Cousins M J, Otte A, et al. Pregabalin in central neuropathic pain associated with spinal cord injury: a placebo-controlled trial [J]. Neurology, 2006, 67: 1792-800.	262	17.47	11

### Year of Publication

The top 100 most-cited papers were published from 2000 (24) to 2017(13). The majority of papers were presented in the 2000s (93%), but only 7% were published in the 2010s. The mean number of citations per paper was 480.72 overall (139.74 in the 2000s and 350.47 in the 2010s).

**Figure 2:**
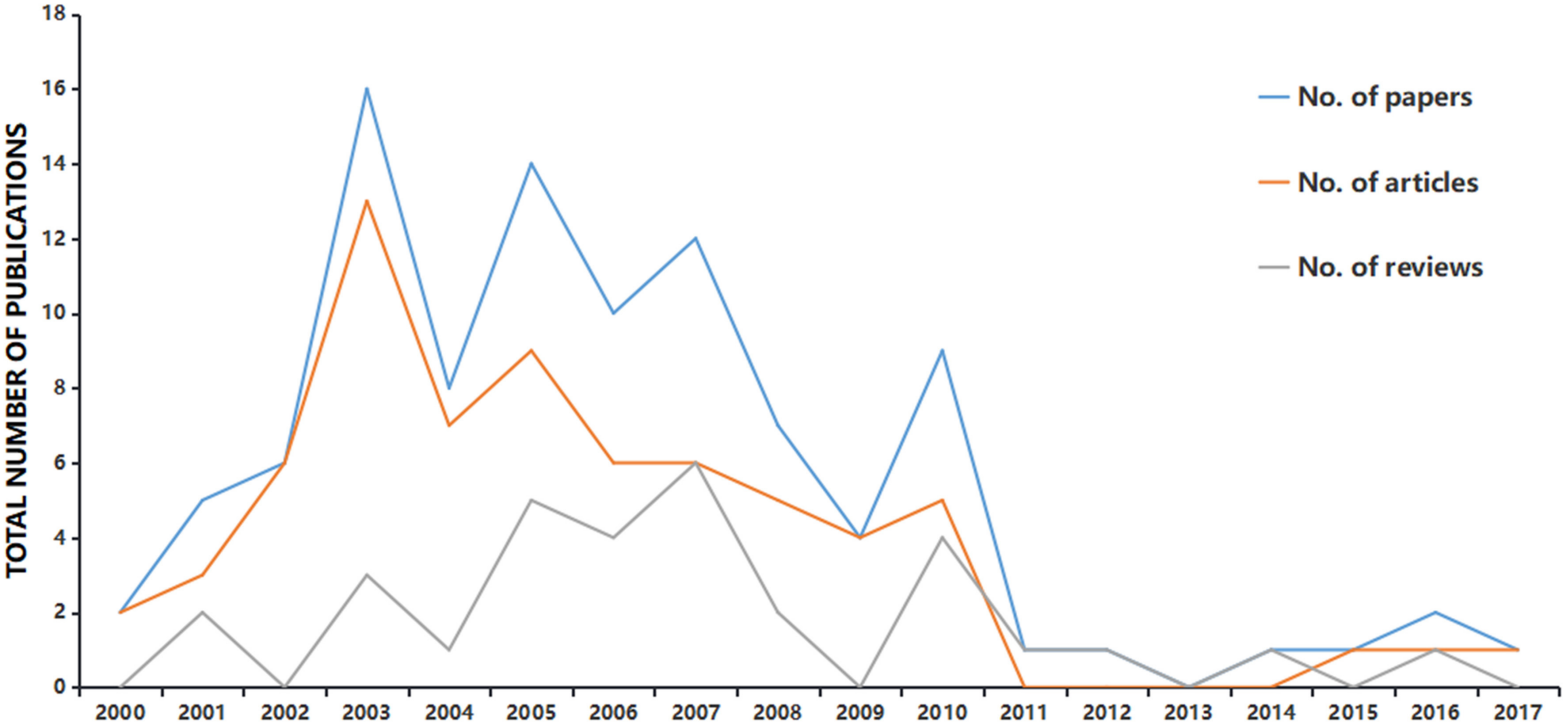
Number of publications among different types of articles according to publication year.

The largest number of papers published in a single year was 16, and this occurred in 2003. 2005 with 14 papers was the second peak. During this period, except for years 2011, 2012, and 2014, the number of articles was generally higher than the number of reviews (Figure 2). Among the top-cited papers, “Spared nerve injury: an animal model of persistent peripheral neuropathic pain” published in 2000 was the earliest cited paper (citations in 2020 = 99) (24). We executed a two-time point analysis to compare the papers before and after 2008 (Table 2). Publications before 2008 were ~4 times that of publications after 2008. However, the total number of citations per paper before 2008 was lower than that after 2008, and the citation difference between the two periods was obvious. Among the top 100 papers related to neuropathic pain, 34% had open access and 14% were highly cited. Notably, all highly cited papers were published after 2008.

**Table 2:** Two-time point analyses comparing journals' top-cited articles before and after 2008.

**Variables**	**Before 2008**	**After 2008**	***P*-value**
Year	2000–2008	2009–2017	
No. of papers	80	20	
Citations WOS per paper	471.71	514.45	0.070
Citations WOS per year	1,797	857.42	0.000
Citations 2020 per paper	24.89	67.05	0.002
Country			
USA	36 (85.71%)	6 (14.29%)	
Canada	8 (72.73%)	3 (27.27%)	
Others	36 (73.47%)	11 (26.53%)	
Document type			
No. of article	57 (82.61%)	12 (17.39%)	
No. of review	23 (74.19%)	8 (25.81%)	
Citations per article	451.21	560.17	0.137
Citations per review	522.52	445.88	0.929
Open access	24 (70.59%)	10 (29.41%)	
Highly cited	0	14 (100%)	
Average IF (2020)	12.468 (2.153–74.699)	13.786 (3.125–60.39)	0.379

### Distribution of Countries and Institutions

**Figure 3:**
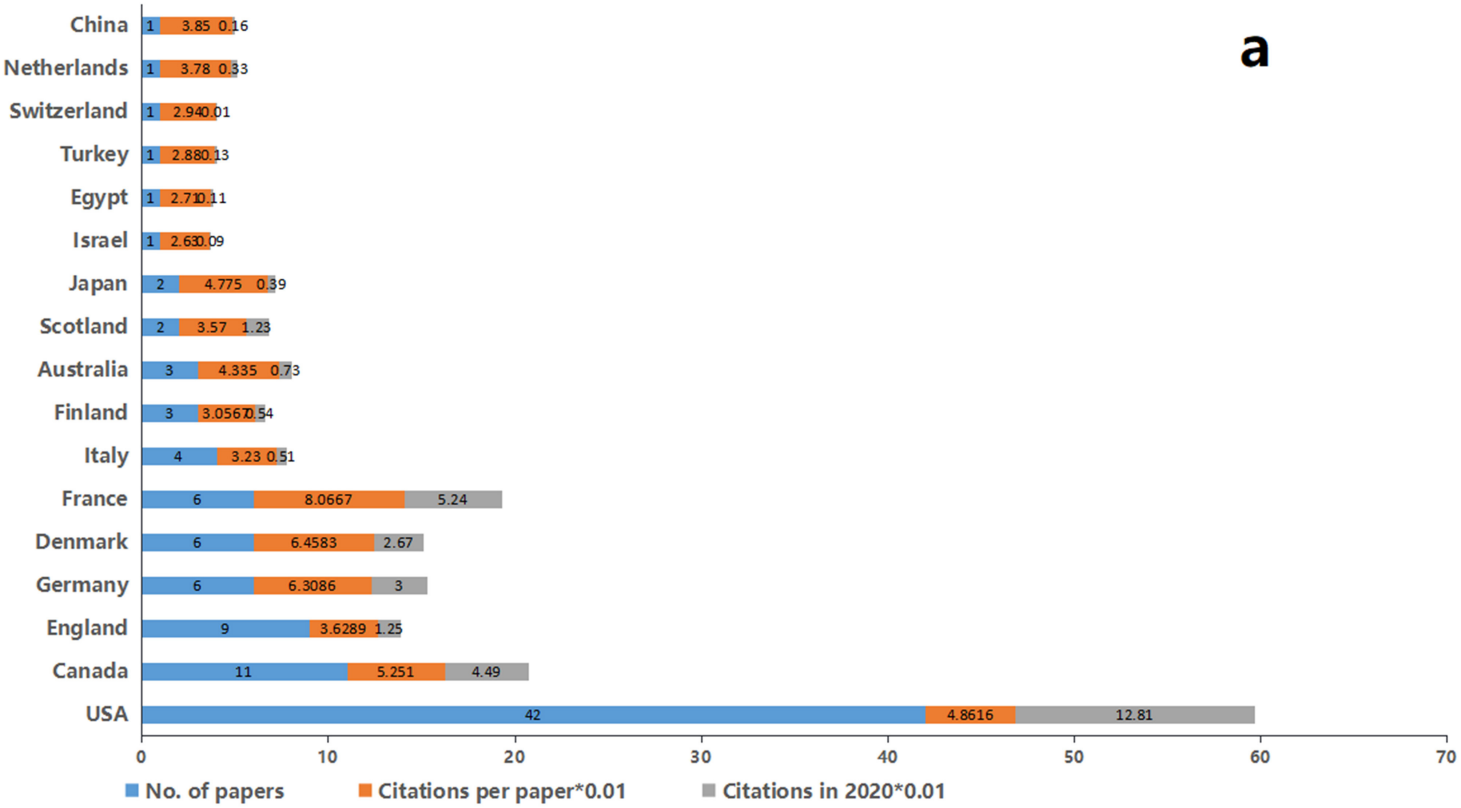
Countries of region of the top 100 most-cited papers.

**Figure 4:**
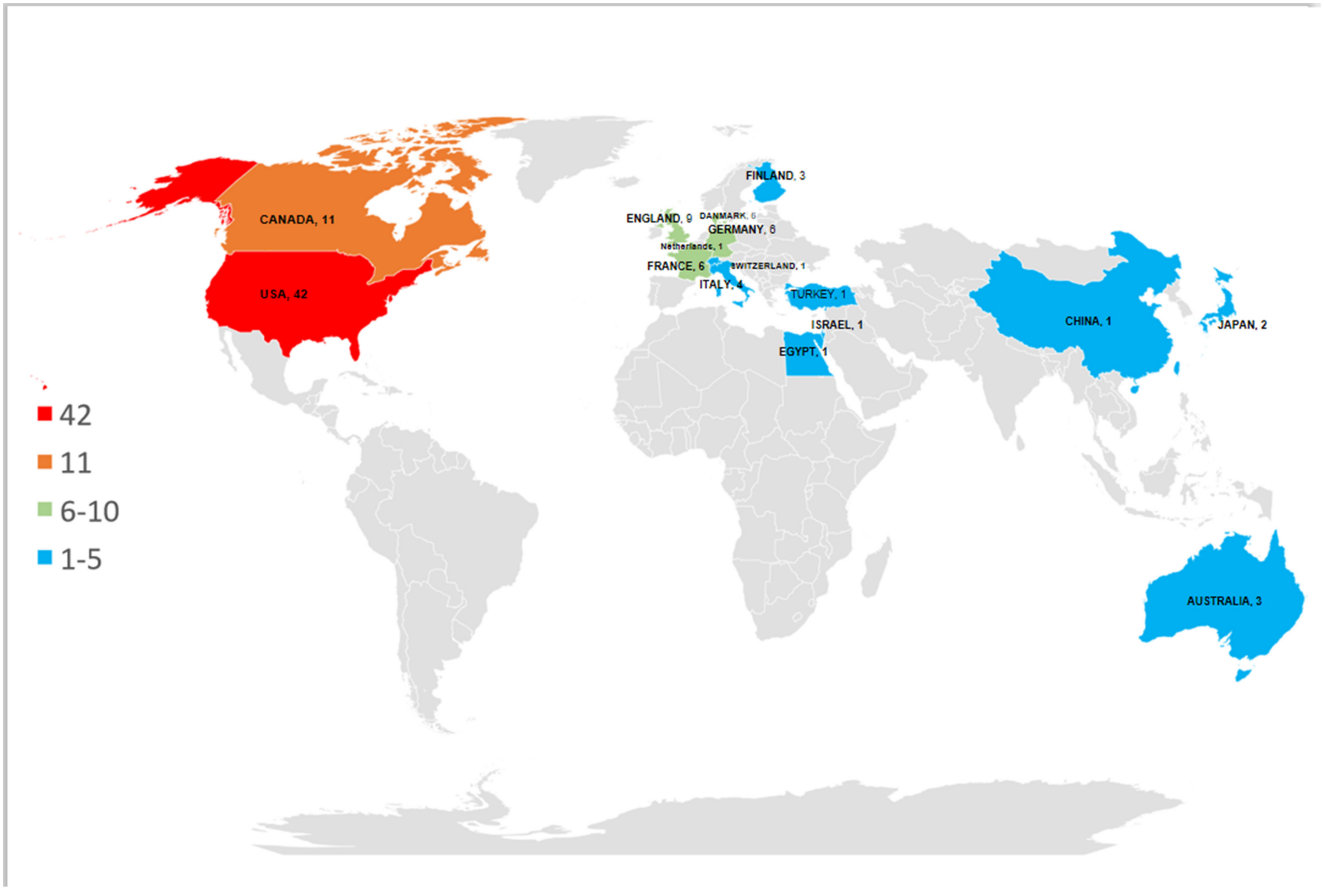
The heat map showing the distribution of the top 100 most-cited papers in world.

The 100 top-cited papers were from 17 countries; the USA and Canada were the most productive in this regard. Nearly half of the papers published (*n* = 42) were from the USA, and around one-tenth (*n* = 11) was from Canada. England with 9 papers ranked third, followed by Germany, France, and Denmark that contributed six papers each to the list (Figure 3). The distribution is demonstrated on a world map (Figure 4). The map shows that a vast majority of publication outputs were from North America and Western Europe. None of the publications included were published in South America.

**Figure 5:**
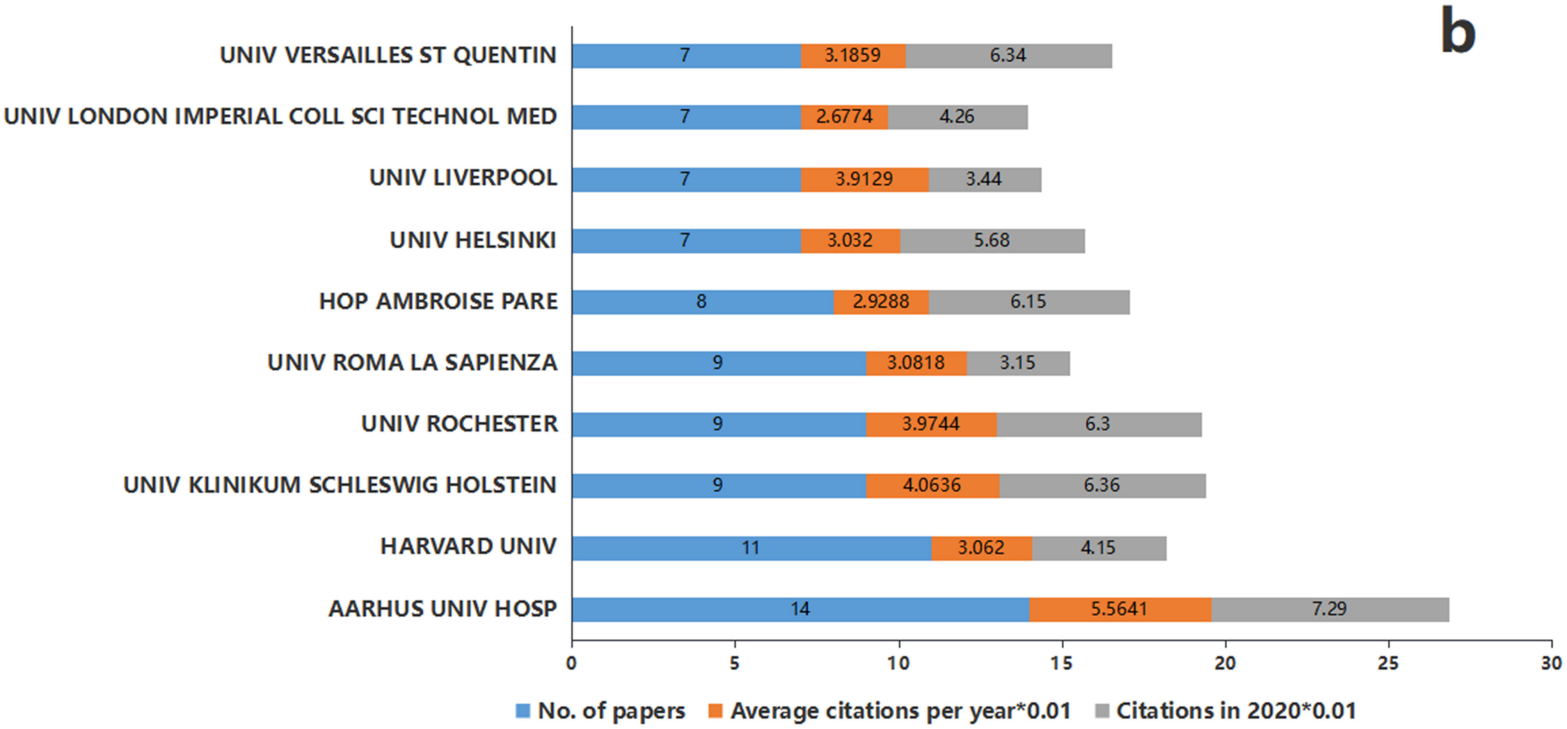
Institutions with at least seven papers in the top 100 most-cited papers.

A total of 259 institutions published at least one top-cited paper, with 139 (53.7%) institutions publishing only one. A small number of institutions accounted for a high proportion of the highest cited papers, similar to the previous findings of lncRNAs studies (25). Figure 5 shows that the top 10 institutions collectively published at least seven papers. Aarhus University Hospital from Denmark topped the list with 14 papers; its output was smaller than that of the US but surpassed that of Canada and England. Aarhus University Hospital was followed by Harvard University from the USA (*n* = 11) and University Klinikum Schleswig Holstein from Germany (*n* = 9).

### Distribution of Journals

**Figure 6:**
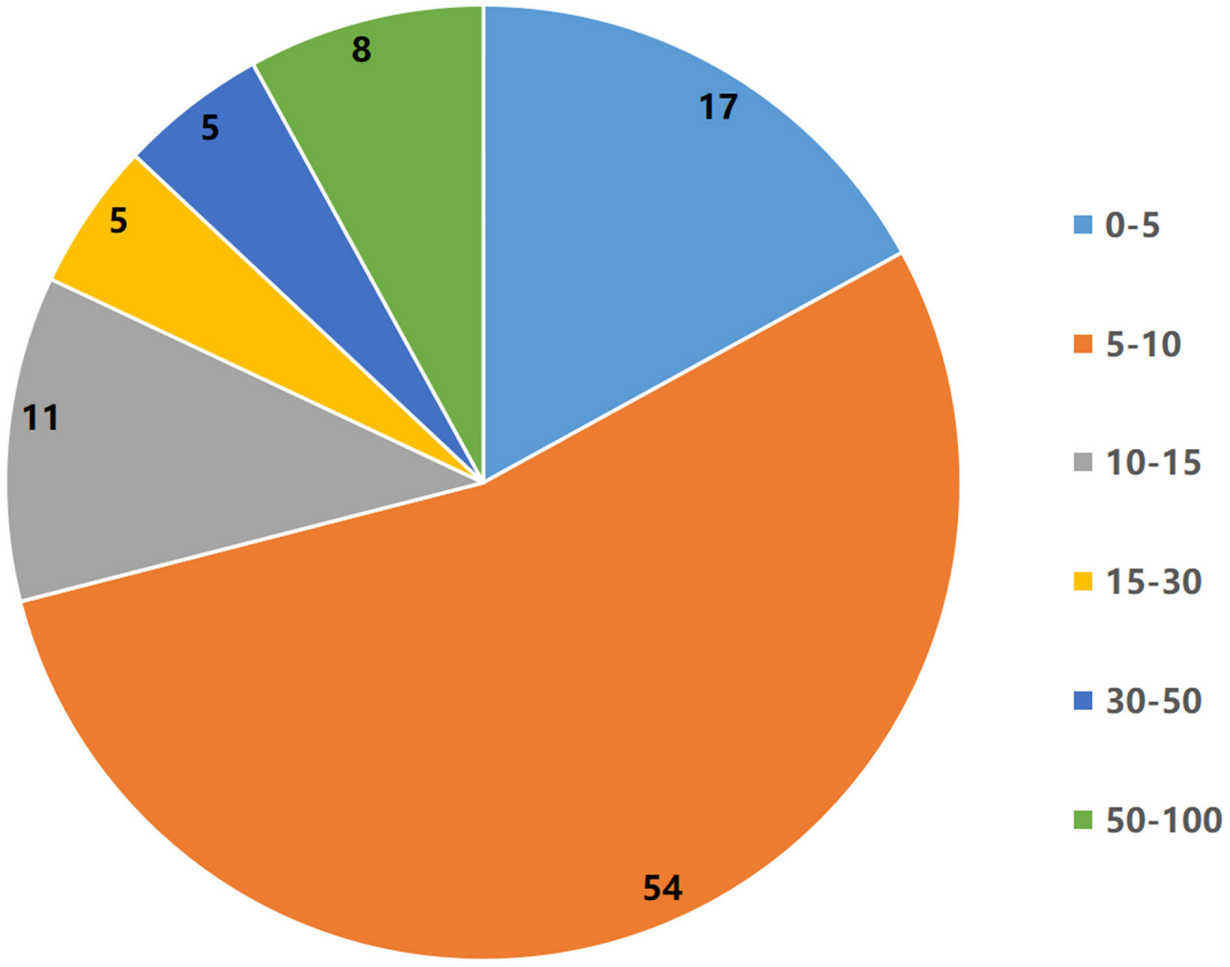
The number of articles corresponding to different impact factors in the top 100 most-cited papers.

Forty-four kinds of journals contributed to the 100 top-cited papers, which were predominantly published in Pain (*n* = 23), followed by the Journal of Neuroscience (*n* = 8), Neurology (*n* = 7), and European Journal of Neurology (*n* = 6). With regard to the average citation number per paper, the Journal of Lancet Neurology ranked first with a mean of 993.0 citations per paper, followed by Nature with a mean of 941.5. All journals containing more than two publications are summarized in Table 3. The IF for journals in the top 100 most-cited papers ranged from 3.037 to 91.245 (Figure 6). The IF of 44 journals participating in the publication of the top 100 most-cited papers ranged from 3.037 to 91.245, in which 71 journals had IF between 3 and 10, 16 journals had IF between 10 and 30, and 13 journals had IF above 30 (indicating a relatively wide reader base). In the top 100 list, paper counts (*r* = 0.272, *P* = 0.009) and citation (*r* = 0.129, *P* = 0.034) counts were significantly related to IF.

**Table 3:** Journals contributed ≥2 papers in the top 100 most cited list.

**Rank**	**Journal**	**Country**	**No. of papers**	**Citations per paper**	**Citations WOS**	**IF (2020)**	**IF (5 year)**	**JCR category**	**JCR partition**
1	Pain	USA	23	578.30	13,301	6.961	7.704	Anesthesiology; Clinical Neurology; Neurosciences	Q1, Q1, Q1
2	Journal of Neuroscience	USA	8	383.75	3,070	6.167	6.993	Neurosciences	Q1
3	Neurology	USA	7	521.14	3,648	9.91	10.663	Clinical Neurology	Q1
4	European Journal of Neurology	USA	6	469.67	2,818	6.089	5.308	Clinical Neurology; Neurosciences	Q1, Q1
5	Proceedings of the National Academy of Sciences of the United States of America	USA	5	365.20	1,826	11.205	12.291	Multidisciplinary Sciences	Q1
6	New England Journal of Medicine	USA	4	701	2,804	91.245	89.666	Medicine, General and Internal	Q1
7	Journal of Pharmacology and Experimental Therapeutics	USA	3	283.67	851	4.03	4.422	Pharmacology and Pharmacy	Q2
8	Clinical Journal of Pain	USA	2	317	634	3.442	4.089	Anesthesiology; Clinical Neurology	Q2, Q2
9	Cochrane Database of Systematic Reviews	USA	2	330	660	9.266	9.871	Medicine, General and Internal	Q1
10	Lancet Neurology	USA	2	993	1,986	44.182	41.51	Clinical Neurology	Q1
11	Nature	England	2	942	1,884	49.962	54.637	Multidisciplinary Sciences	Q1
12	Nature Medicine	USA	2	422	844	53.44	49.248	Biochemistry and Molecular Biology; Cell Biology; Medicine, Research and Experimental	Q1, Q1, Q1
13	Nature Neuroscience	USA	2	702	1,404	24.884	25.875	Neurosciences	Q1
14	Neuron	USA	2	577.5	1,155	17.173	18.658	Neurosciences	Q1

### Distribution of Authors

Table 4 illustrates the most productive writers (those who authored at least seven papers) and their basic research institutions. In total, 582 authors contributed to these works. The largest contributor was Jensen T.S., who published the largest number of publications (*n* = 15); the total number of citations for his papers was 9,793. Baron R. with 14 papers was the next largest contributor. Attal N. and Cruccu G. were tied at the third place with 10 papers each. Cruccu G. from Sapienza University Rome, Dworkin R. H. from University of Rochester, and Finnerup N. B. from Aarhus University were the first author of the four papers in our list.

**Table 4:** Authors with at least seven papers in the top 100 most-cited list.

						**Position on author list**		
**Author**	**No. of papers**	**Citations per paper**	**Citations WOS**	**Citation in 2020**	**Affiliation**	**First-author**	**Correspondent author**	**Other**
Jensen TS	15	652.87	9,793	740	Aarhus University	1	1	13
Baron R	14	619.5	8,673	1,007	Schleswig Holstein University Hospital	2	0	12
Attal N	10	645.4	6,454	742	Hopital Universitaire Ambroise-Pare–APHP	2	1	7
Cruccu G	10	553.6	5,536	407	Sapienza University Rome	4	0	6
Treede RD	8	818	6,544	527	Johannes Gutenberg University	1	0	7
Dworkin RH	7	759.71	5,318	400	University of Rochester	4	0	3
Finnerup NB	7	690.86	4,836	617	Aarhus University	4	0	3
Hansson P	7	799.86	5,599	581	University of Oslo	0	0	7
Ji RR	7	448.71	3,141	213	Harvard Medical School	1	5	1
Rice ASC	7	689.14	4,824	489	Imperial College London	1	1	5

### Distribution of Study Fields

In Supplementary Table 1, the top 100 papers in neuropathic pain are classified into different study fields on the basis of WOS categories. The leading WOS category was “Neurosciences” (*n* = 50), and 46 papers were categorized under “Clinical Neurology.” Many studies were also conducted in other fields, such as “Anesthesiology,” (*n* = 28) “Medicine General,” (*n* = 12), and “Multidisciplinary (*n* = 8).”

### Co-occurrence of Keywords

CiteSpace V was used to extract the keywords of the top 100 papers on neuropathic pain. A network analysis of the author's keywords or subject words was carried out during the publication time of the article, namely, 2000–2017 (Supplementary Figure 1). The results showed that “allodynia,” (*n* = 0.32) “double blind,” (*n* = 0.3) “amitriptyline,” (*n* = 0.24) “management,” (*n* = 0.19), and “mechanism” (*n* = 0.17) had a high degree of centrality during this period. Supplementary Table 2 shows that “neuropathic pain,” (*n* = 30) “double blind,” (*n* = 22), and “post-herpetic neuralgia” (*n* = 22) were the most frequently used keywords.

### Type of Document

In terms of document type, the original articles comprised 69% of the most-cited papers, and the remaining 31% were reviews. Total citations (*P* = 0.011), annual citations (*P* = 0.013), and citation 2020 (*P* = 0.001) significantly differed among the different document types.

## Discussion

We performed a bibliometric study of the top 100 most-cited papers on neuropathic pain worldwide over the last 20 years to help understand academic concerns and the directions of technological innovations in neuropathic pain worldwide. Here, we summarized several characteristics of these papers to understand the history and professional prospects comprehensively.

### Characteristics of the Top 100 Papers

#### Citation Analysis

Many reports on similar citation analysis of other professions and diseases are available. The citation counts for the top 100 most-cited articles on dry eye varied from 96 to 610 times; those on lncRNAs varied from 249 to 2,828 times; and those on back pain varied from 249 to 1,638 times (25–27). Compared with previous citation analyses, the number of citations in this study ranged close to that of the 100 most-cited papers on back pain, which partly reflects the importance and academic attention of neuropathic pain research (26).

How to evaluate a good paper apart from adopting the immeasurable peer review is debatable, but a relatively reasonable index is the number of citations, which varies in different sub-disciplines and depends on the scale of the scientific community (28). In general, an article with 100 or more citations is considered a “classic” based on the research field and may even be a seminal paper (29); thus, new researchers in a special field could read them before conducting further research (30). In the current research, all top 100 papers can be regarded as “classic” papers based on their citation counts, and the citation frequency of the papers is between 262 and 1,569, which is significantly higher than that of other pain symptoms, such as postoperative pain (31) and pediatric pain (19). The findings suggest that neuropathic pain research is a major focus in medicine and health.

The paper that ranked first, which was based on a “redefinition and grading system for clinical and research purposes for neuropathic pain,” had a maximum of 1,569 citations; it was published in Neurology in 2008 and written by Treede et al. (23). To prevent bias due to long-term citations of early papers, we calculated annual citations to assess the relative influence of a publication. Publications with many total citations but few annual citations have vital historical importance in a given period. By contrast, publications with many total and annual citations may be relevant to current research and should be regarded as milestones in true neuropathic pain research. In addition to original articles [e.g., a vaccine to prevent herpes zoster and postherpetic neuralgia in older adults (32)], the top 100 most-cited articles on neuropathic pain includes some consensus and position papers [e.g., neuropathic pain: redefinition and a grading system for clinical and research purposes (23)], guidelines [e.g., EFNS guidelines on the pharmacological treatment of neuropathic pain: 2010 revision (33)], and systematic reviews [e.g., pharmacotherapy for neuropathic pain in adults: a systematic review and meta-analysis (34)]. In general, however, consensus and position papers, guidelines and systematic reviews all receive more citations than original articles, which is a bias that must be taken into account when conducting citation analysis.

The papers published in the last 3 years did not accumulate enough citations to be included in the top 100 list. Hence, we tabulated the top 10 most-cited papers from 2018 to 2020 to show the “rising stars” in the neuropathic pain field (Table 5). We found that the average citations in 2020 for these newly published papers were much higher than those for the most-cited papers published in 2000–2008, indicating the improvement in the quality of research in recent years and the potential academic importance in the future.

**Table 5:** The top 10 most-cited papers on neuropathic pain from 2018 to 2020.

**Rank**	**First author**	**Paper**	**Citations WOS**	**Citations per year**	**Citations in 2020**
1	Inoue K	Inoue Kazuhide, Tsuda Makoto, Microglia in neuropathic pain: cellular and molecular mechanisms and therapeutic potential. [J]. Nat Rev Neurosci, 2018, 19: 138-152.	131	43.67	73
2	Mücke M	Mücke Martin, Phillips Tudor, Radbruch Lukas et al. Cannabis-based medicines for chronic neuropathic pain in adults. [J]. Cochrane Database Syst Rev, 2018, 3: CD012182.	115	38.33	54
3	Alles SRA	Alles Sascha R A, Smith Peter A, Etiology and Pharmacology of Neuropathic Pain. [J]. Pharmacol Rev, 2018, 70: 315-347.	71	23.67	38
4	Sommer C	Sommer Claudia, Leinders Mathias, Üçeyler Nurcan, Inflammation in the pathophysiology of neuropathic pain. [J]. Pain, 2018, 159: 595-602.	67	22.33	42
5	Donvito G	Donvito Giulia, Nass Sara R, Wilkerson Jenny L et al. The Endogenous Cannabinoid System: A Budding Source of Targets for Treating Inflammatory and Neuropathic Pain. [J]. Neuropsychopharmacology, 2018, 43: 52-79.	64	21.33	32
6	Scholz J	Scholz Joachim, Finnerup Nanna B, Attal Nadine et al. The IASP classification of chronic pain for ICD-11: chronic neuropathic pain. [J]. Pain, 2019, 160: 53-59.	63	31.5	43
7	Chen J	Chen Jinjun, Li Lingyong, Chen Shao-Rui et al. The α2δ-1-NMDA Receptor Complex Is Critically Involved in Neuropathic Pain Development and Gabapentin Therapeutic Actions. [J]. Cell Rep, 2018, 22: 2307-2321.	58	19.33	22
8	De Gregorio D	De Gregorio Danilo, McLaughlin Ryan J, Posa Luca et al. Cannabidiol modulates serotonergic transmission and reverses both allodynia and anxiety-like behavior in a model of neuropathic pain. [J]. Pain, 2019, 160: 136-150.	48	24	34
9	Li Y	Li Yan, North Robert Y, Rhines Laurence D et al. DRG Voltage-Gated Sodium Channel 1. 7 Is Upregulated in Paclitaxel-Induced Neuropathy in Rats and in Humans with Neuropathic Pain. [J]. J Neurosci, 2018, 38: 1124-1136.	48	16	22
10	Galor A	Galor Anat, Moein Hamid-Reza, Lee Charity et al. Neuropathic pain and dry eye. [J]. Ocul Surf, 2018, 16: 31-44.	44	14.67	25

#### Year of Publication

In a chronological order, we noticed that although our search spanned the period 2000–2020, the top 100 papers obtained were actually published between 2000 and 2017, and 60% of the papers were published in 2003–2007. Previous analyses have shown that 2003 was the most published year in two decades. Exactly 2,482 papers on neuropathic pain were published from 2018 to 2020, but they were not cited enough to be included in the list of top 100 papers.

Publication date has a significant effect on citation numbers. However, predicting the real influence of a study 20 years after its publication is almost impossible (29, 35, 36). The longer the time that has elapsed after publication, the greater the chance of being quoted is regardless of the impact. This trend has been observed in almost all majors. Our results showed the same trend because 80% of the top 100 papers were published before 2008.

One of the most striking features of this research is the two-time point analysis of papers from before and after 2008. The two-time point analysis showed that the number of papers published after 2008 was much smaller than that before 2008, but the number of citations per paper was larger than that before 2008. This result might be due to the fact that post-2008 papers have improved their quality and relevance to clinical practice and research and have achieved academic importance in the neuropathic pain field. Another possible reason is that the number of articles associated with neuropathic pain has increased in recent years, thereby providing increased citation opportunities. It is worth noting that not only articles related to pain, but also global scientific output have shown explosive growth. The latest study was published in 2017, with 387 citations at present (13); it is followed by an article from 2016, which now has 489 citations (37). This phenomenon could mean that the study of neuropathic pain has been further extended and has deepened in recent years.

In this study, a paper's total citation rate was unrelated to the paper's publication date (*r* = 0.118, *p* = 0.242). However, the papers' current citation rate (as measured by citations in 2020) suggested that papers published after 2008 were likely to be cited in recent years. Such a correlation was statistically significant in this study (*r* = 0.533, *p* < 0.015).

#### Distribution of Countries and Institutions

The geographical distribution of the papers was also demonstrated. Consistent with the findings of several other works, the most top-cited neuropathic pain research was from countries and institutions in two major areas: North America and Western Europe.

The USA, Canada, and England were the three largest contributors (62% of all publications) to the top 100 list.

Aarhus University Hospital and Harvard University were the two largest contributors (25% of all publications) to the top 100 list. The USA with the most publications and citations ranked first in almost every category and was arguably the leader of neuropathic pain research in terms of quality and quantity. This phenomenon is due to the following reasons:

1. Countries with high gross domestic product (GDP), such as the US, Canada, and Denmark, invest heavily in medical research; a previous study has proven that a country's per capita GDP is weakly correlated with its study results (38).
2. There's no doubt that native English speakers get more quotes. For example, American authors are more likely to cite local papers than foreign ones, and their papers are more likely to be published in American journals than foreign ones (16, 39).
3. Mainstream countries dominate publications, and this model has appeared in many medical-related topics, such as lncRNAs (25), human papillomavirus (40), epigenetics (41), and Parkinson's disease (42), thus reflecting the great influence of the USA in the field of medicine.
4. It's worth noting that the citation rate of white and male articles is much higher than that of non-white and female articles (43).

Conversely, we found several papers from Japan and China in Asia, Egypt in Africa, and Australia in Oceania suggesting that neuropathic pain is universal on a global scale. This result again demonstrates that this disease has a research value. Furthermore, this result means that the achievements from these countries and institutions are publicly shared so that numerous people can benefit from them.

#### Distribution of Journals

We noticed that two journals exhibited strong professional attributes and contributed almost a third to the 100 most cited papers. These journals were Pain (*n* = 23) with 13,512 citations in 2020 and whose current IF is 6.961 and the Journal of Neuroscience (*n* = 8) with 3,070 citations in 2020 and whose IF in 2020 was 6.167. In addition to specific journals in this field, several general medical journals, such as The New England Journal of Medicine (IF2020 = 91.245) and Nature Medicine (IF2020 = 53.44), also played an important role in this analysis.

The IFs of 44 journals that participated in the publication of the 100 most frequently cited papers were all >3.0, indicating the dynamism and importance of neuropathic pain research. Ten papers were published in six famous journals with high IF, namely, New England Journal of Medicine, The Lancet, Journal of the American Medical Association, Nature, Science, and Nature Reviews Disease Primers, which are leading journals in science and medicine used to spread cutting-edge research.

The IF value of a journal can be a valid citation predictor. This research supports the theory that the number of papers and citations is positively correlated with journal IF. Moreover, nearly all of the most-cited neuropathic pain papers were published in journals from the USA and England. Given that these famous journals have high rankings and wide influence to attract readers and citations, most authors of high-quality work may prefer their papers to be published in these journals, which in turn maintains the high IF of the journals (44, 45).

#### Distribution of Authors

Several authors were connected to the top-cited publications, and most of them contributed as both the corresponding author and first author. In general, the authors who contributed the most were Jensen T. S. (*n* = 15), Baron R. (*n* = 14), Attal N. (*n* = 10), and Cruccu G. (*n* = 10). This study found that three authors (Cruccu G., Dworkin R. H., and Finnerup N. B.) published 12 papers as first authors, and their papers have been cited 7,517 times in total. This finding highlights how a relatively small number of authors can make substantial contributions to the impact of a journal or research field (46). The three key authors contributed 12% to the first 100 papers, and they accounted for nearly 16% of the citations.

For example, Cruccu G. contributed four studies published in 2007–2010 and was one of the best performing authors. These studies mainly proposed EFNS guidelines on neuropathic pain evaluation and compared the use of different screening tools to classify neuropathic pain (47, 48). Diagnostic testing methods are often described within the framework of neuropathic pain papers and are thus frequently cited. Our analysis revealed that Treede's article entitled “Neuropathic pain–Redefinition and grading system for clinical and research purposes” was the most cited paper (*n* = 1,569) (23). The study provided a precise definition of neuropathic pain (i.e., a direct result of an injury or disease that affects the somatosensory system). A comprehensive neuropathic pain classification system was also proposed for clinical and study goals.

All of these authors must be recognized as important contributors to the neuropathic pain field. However, compared with the top articles in other popular fields, such as cancer, Parkinson's disease, and orthopedics, a gap still exists. Therefore, authors should strive to carry out high-quality research while attempting to increase the quantity of their works.

#### Study Hotpots

Among the 100 most frequently cited papers, more than half were linked with neuroscience research directions, including neuroscience (*n* = 50), clinical neurology (*n* = 46), and anesthesiology (*n* = 28). The diagnosis and treatment of neuropathic pain have developed rapidly in the last few decades. Neuropathic pain research is distributed worldwide in neuroscience in the life sciences and neurology in clinical medicine due to the neural basis of the neuropathic pain mechanism. In our study, the topic with the highest frequency of occurrence was the pathology, pathogenesis, and drug therapy of neuropathic pain (*n* = 71), reflecting the high study interest in this subject in the twenty-first century. Most of these papers involved drug therapy (34, 49) (*n* = 25), followed by post-herpetic neuralgia (32, 50) (*n* = 6) and trigeminal neuralgia (51) (*n* = 4). On the basis of these studies and their results, neuropathic pain has been classified accurately, and several key mechanisms, including abnormal discharging of nociceptive nerves, peripheral, and central sensitization, and impaired inhibitory modulation, have been established (21). The proportion of studies involving “quality of life and/or patient-reported results” (*n* = 3) was extremely low (52). Nevertheless, with the recent extensive attention paid to these topics in medical and neuroscience literature, publications might fall into these categories in future versions of this analysis.

The citations of a paper often follow a time process. The longevity of papers shows the impact of papers on scientific research. In the last year (2020), 17% of the top-cited papers were cited fewer than 10 times, which might indicate that the content of these papers is no longer the current study hotpots.

### Distribution of Keywords

Keywords with citation bursts can reflect the development of a knowledge field. Our keyword centrality analysis showed that allodynia (*n* = 0.32), double blind (*n* = 0.3), amitriptyline (*n* = 0.24), management (*n* = 0.19), and mechanism (*n* = 0.17) had high centrality in the past 20 years, indicating that these research directions are essential, which is consistent with the research results on keywords. It is also consistent with the emphasis placed on diagnosis in the 1990s. Several differences were observed between the 2010s and the 2000s. In the 2010s, the keywords that appeared frequently were quality of life, pharmacological treatment, concentration capsaicin patch, and other related words. This observation suggests that researchers have begun to expand their research to several treatments and pay increasing attention to prognostic assessments, new treatment techniques, quality of life, and other aspects of neuropathic pain therapy. Gradually, neuropathic pain research has begun to blossom. These results also indicate that neuropathic pain is still a disease that needs to be solved urgently, and the deficiencies and innovations in this field, such as new drug therapies, pathological mechanisms, and strengthened pain management, must be explored to improve the quality of life.

### Citation Bias

The citation counts that a publication receives can be a useful indicator of a paper's overall impact on the scientific field. However, the “highest citation” efforts, such as the current research, do not fully reflect the impact of a publication (46). Furthermore, citation analyses could be influenced by many factors, such as date of publication, research topic, and document type. Thus, incorrect, inaccurate, or exaggerated citation counts may be identified in citation analyses.

First, most journals do not have open access and instead adopt a pay-per-view scheme. Thus, not everyone can access them successfully. Second, past studies have shown that a paper is likely to be forgotten as time passes. Moreover, over time, even “citation classics” are cited less frequently than before because their content is incorporated into general medical and neuroscience knowledge; this phenomenon is known as “obliteration by incorporation” (53). Therefore, the ranking of the most-cited papers fluctuates over time. Third, the recent popularity of open-access journals has changed the impact of time or paper longevity on citations.

According to reports, open-access articles usually have numerous citations (54). Specifically, in this study, top-cited papers published after 2008 received three times as many citations per article in 2020 as papers published before 2008, even though their life spans were much shorter.

The fourth limitation is that the basis for selecting papers may vary; different selection criteria may result in a different list. When the number of citations is used as a measure to quantify a paper's influence in bibliometrics, the most-cited paper is usually considered a “citation classic” (39).

Lastly, although the authors, journals, and countries listed in the different tables published the most-cited papers, their contributions and the mechanisms that produce the most-cited papers vary widely.

In future evaluation studies, a multi-indicator cluster survey can be used in conjunction with other indicators to effectively describe the role and contribution of individuals or organizations in cooperation.

### Strengths and Limitations

One of the advantages of this study is that it fully considered the inherent time deviation of bibliometrics. We performed a two-time analysis before and after 2008 and summed up the 10 most-cited publications from 2018 to 2020. This sub-analysis does not reflect the overall trend because actual paper citations may change significantly over time. Then, we identified the authors (e.g., first author and affiliation), journals (e.g., country of origin, IF, and JCR category), and most popular research categories. Lastly, we performed statistical analyses to determine the underlying factors that may be related to citation counts.

Although our results provide some valuable information, the limitations of this research need to be recognized. First, the search strategy depends only on the relevant search terms contained in the title or keywords. We may have overlooked several popular publications, such as those indexed by “low back pain” (55). Second, the language of the papers in WOS is restricted to English; thus, studies written in other languages may have been omitted. Japan, for instance, has many influential researchers whose low output may be partly due to their preference for publication in their native language. Third, paper with fewer than 200 citation times were excluded to reduce the number of papers that required follow-up screening, which might miss clinically important articles. Finally, several papers obtained from WOS may be delayed, resulting in flawed citations. When articles are screened by the number of citations, new publications in the field that are of great significance but have not yet reached a high citation level tend to be overlooked.

## Conclusion

Neuropathic pain research has grown impressively in the past two decades, as evidenced by improvements in research quality and increments in the number of research papers. This bibliometric research is the first to identify and discuss the 100 most-cited papers published in the field of neuropathic pain. Despite the study's limitations, by reviewing these top papers, researchers can immediately understand the significant progress neuropathic pain has achieved over the past two decades, and targeted scientific questions can be selected to fill the gap in research.

This analysis provides insights from prominent individuals and institutions who have contributed considerably to neuropathic pain research and identifies characteristics that are relevant to high citation counts. The citation frequency of the top 100 papers published from 2000 to 2017 ranged from 262 to 1,569. IF and country or region were found to be closely associated with citations. Most of the papers had much higher citations in 2020 compared with their annual citations, reflecting the fact that the top 100 papers have received the continuous attention of researchers and may be of potential academic importance in the future. The two-time point analysis indicated that the citation longevity and citation modes of the most-cited papers changed over time. Compared with the early most-cited papers, recently published most-cited papers reached the peak citation rate faster. The exploration of drug therapy, pathophysiological mechanism, diagnosis, and screening tools of neuropathic pain might be the research focus in the future. Overall, the neuropathic pain field appears to be promising. The major contributions of the most influential studies can serve as an important reference for all pain physicians and neuroscientists because their goal is to improve clinical practice and their own scientific findings.

## Data Availability Statement

The original contributions presented in the study are included in the article/Supplementary Material, further inquiries can be directed to the corresponding authors.

## Author Contributions

X-QW: conceptualization, investigation, resources, and funding acquisition. H-YX: methodology, software, formal analysis, data curation, writing—original draft preparation, visualization, and project administration. HL: validation. X-QW and HL: writing—review and editing and supervision. All authors have read and agreed to the published version of the manuscript.

## Funding

This work was supported by Fok Ying-Tong Education Foundation of China (161092), the Scientific and Technological Research Program of the Shanghai Science and Technology Committee (Fund number: 19080503100), and the Shanghai Key Lab of Human Performance (Shanghai University of Sport) (11DZ2261100).

## Conflict of Interest

The authors declare that the research was conducted in the absence of any commercial or financial relationships that could be construed as a potential conflict of interest.

## Publisher's Note

All claims expressed in this article are solely those of the authors and do not necessarily represent those of their affiliated organizations, or those of the publisher, the editors and the reviewers. Any product that may be evaluated in this article, or claim that may be made by its manufacturer, is not guaranteed or endorsed by the publisher.

## References

[1] Raja SN Carr DB Cohen M Finnerup NB Flor H Gibson S . The revised International Association for the study of pain definition of pain: concepts, challenges, and compromises. Pain. (2020) 161:1976–82. 10.1097/j.pain.0000000000001939 32694387 PMC7680716

[2] Jensen TS Baron R Haanpaa M Kalso E Loeser JD Rice ASC . A new definition of neuropathic pain. Pain. (2011) 152:2204–5. 10.1016/j.pain.2011.06.017 21764514

[3] van Hecke O Austin SK Khan RA Smith BH Torrance N. Neuropathic pain in the general population: a systematic review of epidemiological studies. Pain. (2014) 155:654–62. 10.1016/j.pain.2013.11.013 24291734

[4] Raskin J Pritchett YL Wang F D'Souza DN Waninger AL Iyengar S . A double-blind, randomized multicenter trial comparing duloxetine with placebo in the management of diabetic peripheral neuropathic pain. Pain Med. (2005) 6:346–56. 10.1111/j.1526-4637.2005.00061.x 16266355

[5] Cruccu G Gronseth G Alksne J Argoff C Brainin M Burchiel K . AAN-EFNS guidelines on trigeminal neuralgia management. Eur J Neurol. (2008) 15:1013–28. 10.1111/j.1468-1331.2008.02185.x 18721143

[6] Peul WC van Houwelingen HC van den Hout WB Brand R Eekhof JA Tans JT . Surgery versus prolonged conservative treatment for sciatica. N Engl J Med. (2007) 356:2245–56. 10.1056/NEJMoa064039 17538084

[7] Gilron I Baron R Jensen T. Neuropathic pain: principles of diagnosis and treatment. Mayo Clin Proc. (2015) 90:532–45. 10.1016/j.mayocp.2015.01.018 25841257

[8] Mayoral V Perez-Hernandez C Muro I Leal A Villoria J Esquivias A. Diagnostic accuracy of an identification tool for localized neuropathic pain based on the IASP criteria. Curr Med Res Opin. (2018) 34:1465–73. 10.1080/03007995.2018.1465905 29661030

[9] Xiong HY Zhang ZJ Wang XQ. Bibliometric analysis of research on the comorbidity of pain and inflammation. Pain Res Manag. (2021) 2021:6655211. 10.1155/2021/6655211 33680225 PMC7904349

[10] Zheng K Chen C Yang S Wang X. Aerobic exercise attenuates pain sensitivity: an event-related potential study. Front Neurosci. (2021) 15:735470. 10.3389/fnins.2021.735470 34630022 PMC8494006

[11] Wu B Zhou L Chen C Wang J Hu L Wang X. Effects of Exercise-induced hypoalgesia and its neural mechanisms. Med Sci Sports Exerc. (2021). 10.1249/MSS.0000000000002781. [Epub ahead of print]. 34468414

[12] Finnerup NB Haroutounian S Kamerman P Baron R Bennett DLH Bouhassira D . Neuropathic pain: an updated grading system for research and clinical practice. Pain. (2016) 157:1599–606. 10.1097/j.pain.0000000000000492 27115670 PMC4949003

[13] Colloca L Ludman T Bouhassira D Baron R Dickenson AH Yarnitsky D . Neuropathic pain. Nat Rev Dis Primers. (2017) 3:17002. 10.1038/nrdp.2017.2 28205574 PMC5371025

[14] Schou WS Ashina S Amin FM Goadsby PJ Ashina M. Calcitonin gene-related peptide and pain: a systematic review. J Headache Pain. (2017) 18:34. 10.1186/s10194-017-0741-2 28303458 PMC5355411

[15] Garfield E. 100 citation classics from the Journal of the American Medical Association. JAMA. (1987) 257:52–9. 3537352

[16] Baltussen A Kindler CH. Citation classics in critical care medicine. Intensive Care Med. (2004) 30:902–10. 10.1007/s00134-004-2195-7 14985952

[17] Norton PJ Asmundson GJG Norton RG Craig KD. Growing pain: 10-year research trends in the study of chronic pain and headache. Pain. (1999) 79:59–65. 10.1016/S0304-3959(98)00149-3 9928777

[18] Robert C Caillieux N Wilson CS Gaudy JF Arreto CD. World orofacial pain research production: a bibliometric study (2004-2005). J Orofac Pain. (2008) 22:181–9. 10.1111/j.1365-2842.2007.01816.x 18780531

[19] Caes L Boerner KE Chambers CT Campbell-Yeo M Stinson J Birnie KA . A comprehensive categorical and bibliometric analysis of published research articles on pediatric pain from 1975 to 2010. Pain. (2016) 157:302–13. 10.1097/j.pain.0000000000000403 26529270

[20] Chen YM Wang XQ. Bibliometric analysis of exercise and neuropathic pain research. J Pain Res. (2020) 13:1533–45. 10.2147/JPR.S258696 32612381 PMC7323814

[21] Ye J Ding H Ren J Xia Z. The publication trend of neuropathic pain in the world and China: a 20-years bibliometric analysis. J Headache Pain. (2018) 19:110. 10.1186/s10194-018-0941-4 30442089 PMC6755566

[22] Chen C. Searching for intellectual turning points: progressive knowledge domain visualization. Proc Natl Acad Sci USA. (2004) 101(Suppl 1):5303–10. 10.1073/pnas.0307513100 PMC387312 14724295

[23] Treede RD Jensen TS Campbell JN Cruccu G Dostrovsky JO Griffin JW . Neuropathic pain: redefinition and a grading system for clinical and research purposes. Neurology. (2008) 70:1630–5. 10.1212/01.wnl.0000282763.29778.59 18003941

[24] Decosterd I Woolf CJ. Spared nerve injury: an animal model of persistent peripheral neuropathic pain. Pain. (2000) 87:149–58. 10.1016/S0304-3959(00)00276-1 10924808

[25] Peng MS Chen CC Wang J Zheng YL Guo JB Song G . The top 100 most-cited papers in long non-coding RNAs: a bibliometric study. Cancer Biol Ther. (2021) 22:40–54. 10.1080/15384047.2020.1844116 33315532 PMC7836983

[26] Huang W Wang L Wang B Yu L Yu X. Top 100 cited articles on back pain research: a citation analysis. Spine. (2016) 41:1683–92. 10.1097/BRS.0000000000001736 27798556

[27] Schargus M Kromer R Druchkiv V Frings A. The top 100 papers in dry eye - a bibliometric analysis. Ocul Surf. (2018) 16:180–90. 10.1016/j.jtos.2017.09.006 28923504

[28] Gondivkar SM Sarode SC Gadbail AR Gondivkar RS Chole R Sarode GS. Bibliometric analysis of 100 most cited articles on oral submucous fibrosis. J Oral Pathol Med. (2018) 47:781–7. 10.1111/jop.12742 29905986

[29] Feijoo JF Limeres J Fernandez-Varela M Ramos I Diz P. The 100 most cited articles in dentistry. Clin Oral Investig. (2014) 18:699–706. 10.1007/s00784-013-1017-0 23771182

[30] Zhai X Zhao J Wang Y Wei X Li G Yang Y . Bibliometric analysis of global scientific research on lncRNA: a swiftly expanding trend. Biomed Res Int. (2018) 2018:7625078. 10.1155/2018/7625078 29992161 PMC5994307

[31] Garcia JBS de Moraes EB Neto JOB. A bibliometric analysis of published literature in postoperative pain in elderly patients in low- and middle-income countries. J Clin Med. (2021) 10:2334. 10.3390/jcm10112334 34071737 PMC8198345

[32] Oxman MN Levin MJ Johnson GR Schmader KE Straus SE Gelb LD . A vaccine to prevent herpes zoster and postherpetic neuralgia in older adults. N Engl J Med. (2005) 352:2271–84. 10.1056/NEJMoa051016 15930418

[33] Attal N Cruccu G Baron R Haanpaa M Hansson P Jensen TS . EFNS guidelines on the pharmacological treatment of neuropathic pain: 2010 revision. Eur J Neurol. (2010) 17:1113–e1188. 10.1111/j.1468-1331.2010.02999.x 20402746

[34] Finnerup NB Attal N Haroutounian S McNicol E Baron R Dworkin RH . Pharmacotherapy for neuropathic pain in adults: a systematic review and meta-analysis. Lancet Neurol. (2015) 14:162–73. 10.1016/S1474-4422(14)70251-0 25575710 PMC4493167

[35] Baltussen A Kindler CH. Citation classics in anesthetic journals. Anesth Analg. (2004) 98:443–51. 10.1213/01.ANE.0000096185.13474.0A 14742385

[36] Ahmad P Dummer PMH Noorani TY Asif JA. The top 50 most-cited articles published in the International Endodontic Journal. Int Endod J. (2019) 52:803–18. 10.1111/iej.13083 30667524

[37] Forero M Adhikary SD Lopez H Tsui C Chin KJ. The erector spinae plane block: a novel analgesic technique in thoracic neuropathic pain. Reg Anesth Pain Med. (2016) 41:621–7. 10.1097/AAP.0000000000000451 27501016

[38] Tao T Zhao X Lou J Bo L Wang F Li J . The top cited clinical research articles on sepsis: a bibliometric analysis. Crit Care. (2012) 16:R110. 10.1186/cc11401 22731930 PMC3580668

[39] Ahmad SS Evangelopoulos DS Abbasian M Roder C Kohl S. The hundred most-cited publications in orthopaedic knee research. J Bone Joint Surg Am. (2014) 96:e190. 10.2106/JBJS.N.00029 25410518

[40] Lin HW Yu TC Ho YS. A systemic review of human papillomavirus studies: global publication comparison and research trend analyses from 1993 to 2008. Eur J Gynaecol Oncol. (2011) 32:133–40. 21614898

[41] Lu K Yu S Sun D Xing H An J Kong C . Scientometric analysis of SIRT6 studies. Med Sci Monit. (2018) 24:8357–71. 10.12659/MSM.913644 30457131 PMC6256847

[42] Li T Ho YS Li CY. Bibliometric analysis on global Parkinson's disease research trends during 1991-2006. Neurosci Lett. (2008) 441:248–52. 10.1016/j.neulet.2008.06.044 18582532

[43] Nosek BA Graham J Lindner NM Kesebir S Hawkins CB Hahn C . Cumulative and career-stage citation impact of social-personality psychology programs and their members. Pers Soc Psychol Bull. (2010) 36:1283–300. 10.1177/0146167210378111 20668215

[44] Callaham M Wears RL Weber E. Journal prestige, publication bias, and other characteristics associated with citation of published studies in peer-reviewed journals. JAMA. (2002) 287:2847–50. 10.1001/jama.287.21.2847 12038930

[45] Garfield E. The history and meaning of the journal impact factor. JAMA. (2006) 295:90–3. 10.1001/jama.295.1.90 16391221

[46] Chou CY Chew SS Patel DV Ormonde SE McGhee C. Publication and citation analysis of the Australian and New Zealand Journal of Ophthalmology and Clinical and Experimental Ophthalmology over a 10-year period: the evolution of an ophthalmology journal. Clin Exp Ophthalmol. (2009) 37:868–73. 10.1111/j.1442-9071.2009.02191.x 20092596

[47] Cruccu G Aziz TZ Garcia-Larrea L Hansson P Jensen TS Lefaucheur JP . EFNS guidelines on neurostimulation therapy for neuropathic pain. Eur J Neurol. (2007) 14:952–70. 10.1111/j.1468-1331.2007.01916.x 17718686

[48] Cruccu G Sommer C Anand P Attal N Baron R Garcia-Larrea L . EFNS guidelines on neuropathic pain assessment: revised 2009. Eur J Neurol. (2010) 17:1010–8. 10.1111/j.1468-1331.2010.02969.x 20298428

[49] Finnerup NB Sindrup SH Jensen TS. The evidence for pharmacological treatment of neuropathic pain. Pain. (2010) 150:573–81. 10.1016/j.pain.2010.06.019 20705215

[50] Sabatowski R Galvez R Cherry DA Jacquot F Vincent E Maisonobe P . Pregabalin reduces pain and improves sleep and mood disturbances in patients with post-herpetic neuralgia: results of a randomised, placebo-controlled clinical trial. Pain. (2004) 109:26–35. 10.1016/j.pain.2004.01.001 15082123

[51] Gronseth G Cruccu G Alksne J Argoff C Brainin M Burchiel K . Practice parameter: the diagnostic evaluation and treatment of trigeminal neuralgia (an evidence-based review): report of the Quality Standards Subcommittee of the American Academy of Neurology and the European Federation of Neurological Societies. Neurology. (2008) 71:1183–90. 10.1212/01.wnl.0000326598.83183.04 18716236

[52] Jensen MP Chodroff MJ Dworkin RH. The impact of neuropathic pain on health-related quality of life: review and implications. Neurology. (2007) 68:1178–82. 10.1212/01.wnl.0000259085.61898.9e 17420400

[53] Paladugu R Schein M Gardezi S Wise L. One hundred citation classics in general surgical journals. World J Surg. (2002) 26:1099–105. 10.1007/s00268-002-6376-7 12209239

[54] Larsen PO von Ins M. The rate of growth in scientific publication and the decline in coverage provided by Science Citation Index. Scientometrics. (2010) 84:575–603. 10.1007/s11192-010-0202-z 20700371 PMC2909426

[55] Murray MR Wang T Schroeder GD Hsu WK. The 100 most cited spine articles. Eur Spine J. (2012) 21:2059–69. 10.1007/s00586-012-2303-2 22526702 PMC3463701

