# Supplementary material for: Top 100 Most-Cited Papers in Neuropathic Pain From 2000 to 2020: A Bibliometric Study
Source: Front Neurol. 2021 Nov 12;12:765193. doi: 10.3389/fneur.2021.765193 (PMC8632696; doi:10.3389/fneur.2021.765193)

Supplementary Figure 1. The analysis of keywords. Network map of keywords with the most occurrences from the top 100 most-cited papers on neuropathic pain research.

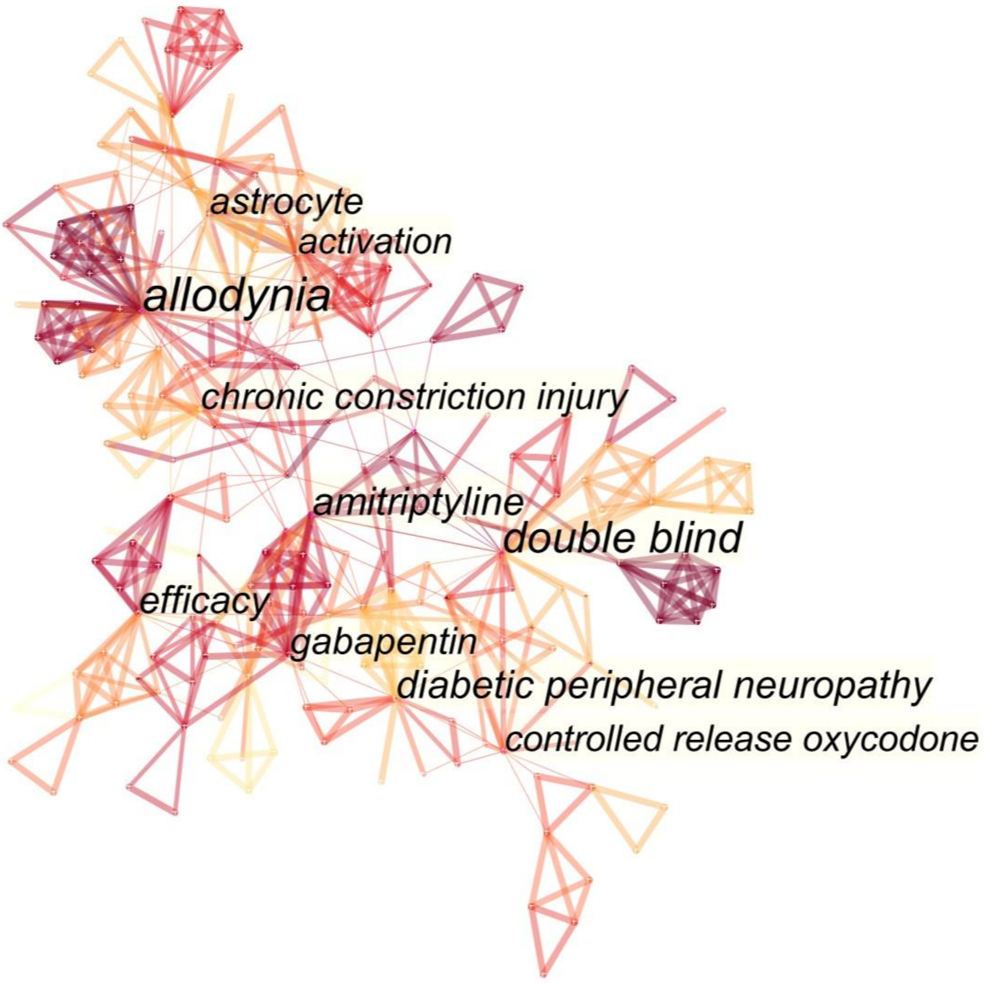

Supplement: Supplementary file 1 [file Image_1.pdf]
