# Supplementary material for: Top 100 Most-Cited Papers in Neuropathic Pain From 2000 to 2020: A Bibliometric Study
Source: Front Neurol. 2021 Nov 12;12:765193. doi: 10.3389/fneur.2021.765193 (PMC8632696; doi:10.3389/fneur.2021.765193)
Supplement: Supplementary file 2 [file Table_1.docx]

**Supplementary table 1. Research fields of the top 100 most-cited list.**

| **Rank** | **Research field** | **No. of papers** | **Citations per paper** | **Citations WOS** |
| --- | --- | --- | --- | --- |
| 1 | NEUROSCIENCES | 50 | 507.22 | 25361 |
| 2 | CLINICAL NEUROLOGY | 46 | 547.5 | 25185 |
| 3 | ANESTHESIOLOGY | 28 | 544.57 | 15248 |
| 4 | MEDICINE GENERAL INTERNAL | 12 | 467.58 | 5611 |
| 5 | MULTIDISCIPLINARY SCIENCES | 8 | 514 | 4112 |
| 6 | PHARMACOLOGY PHARMACY | 7 | 289.57 | 2027 |
| 7 | BIOCHEMISTRY MOLECULAR BIOLOGY | 5 | 337.6 | 1688 |
| 8 | CELL BIOLOGY | 3 | 371 | 1113 |
| 9 | SURGERY | 3 | 299.33 | 898 |
| 10 | IMMUNOLOGY | 2 | 342 | 684 |
