# Supplementary material for: Top 100 Most-Cited Papers in Neuropathic Pain From 2000 to 2020: A Bibliometric Study
Source: Front Neurol. 2021 Nov 12;12:765193. doi: 10.3389/fneur.2021.765193 (PMC8632696; doi:10.3389/fneur.2021.765193)
Supplement: Supplementary file 3 [file Table_2.docx]

**Supplementary table 2. Key words with at least six papers in the top 100 most-cited list.**

| **Key word** | **No. of papers** | **Centrality** |
| --- | --- | --- |
| Neuropathic pain | 30 | 0.08 |
| Double blind | 22 | 0.3 |
| Postherpetic neuralgia | 22 | 0.04 |
| Allodynia | 15 | 0.32 |
| Efficacy | 11 | 0.13 |
| Peripheral nerve injury | 9 | 0.09 |
| Diabetic peripheral neuropathy | 9 | 0.07 |
| Hyperalgesia | 9 | 0.06 |
| Diabetic neuropathy | 9 | 0.01 |
| Amitriptyline | 8 | 0.24 |
| Nerve injury | 8 | 0.04 |
| Placebo controlled trial | 8 | 0.01 |
| Randomized controlled trial | 8 | 0 |
| Spinal cord injury | 8 | 0 |
| Pain | 7 | 0.07 |
| Sensory neuron | 7 | 0.02 |
| Chronic constriction injury | 6 | 0.09 |
| Expression | 6 | 0.07 |
| Dorsal root ganglion | 6 | 0.03 |
| Spinal cord | 6 | 0.02 |
| Trigeminal neuralgia | 6 | 0 |
